# Selective Processing and Metabolism of Disease-Causing Mutant Prion Proteins

**DOI:** 10.1371/journal.ppat.1000479

**Published:** 2009-06-19

**Authors:** Aarthi Ashok, Ramanujan S. Hegde

**Affiliations:** Cell Biology and Metabolism Program, Eunice Kennedy Shriver National Institute of Child Health and Human Development, National Institutes of Health, Bethesda, Maryland, United States of America; University of Edinburgh, United Kingdom

## Abstract

Prion diseases are fatal neurodegenerative disorders caused by aberrant metabolism of the cellular prion protein (PrP^C^). In genetic forms of these diseases, mutations in the globular C-terminal domain are hypothesized to favor the spontaneous generation of misfolded PrP conformers (including the transmissible PrP^Sc^ form) that trigger downstream pathways leading to neuronal death. A mechanistic understanding of these diseases therefore requires knowledge of the quality control pathways that recognize and degrade aberrant PrPs. Here, we present comparative analyses of the biosynthesis, trafficking, and metabolism of a panel of genetic disease-causing prion protein mutants in the C-terminal domain. Using quantitative imaging and biochemistry, we identify a misfolded subpopulation of each mutant PrP characterized by relative detergent insolubility, inaccessibility to the cell surface, and incomplete glycan modifications. The misfolded populations of mutant PrPs were neither recognized by ER quality control pathways nor routed to ER-associated degradation despite demonstrable misfolding in the ER. Instead, mutant PrPs trafficked to the Golgi, from where the misfolded subpopulation was selectively trafficked for degradation in acidic compartments. Surprisingly, selective re-routing was dependent not only on a mutant globular domain, but on an additional lysine-based motif in the highly conserved unstructured N-terminus. These results define a specific trafficking and degradation pathway shared by many disease-causing PrP mutants. As the acidic lysosomal environment has been implicated in facilitating the conversion of PrP^C^ to PrP^Sc^, our identification of a mutant-selective trafficking pathway to this compartment may provide a cell biological basis for spontaneous generation of PrP^Sc^ in familial prion disease.

## Author Summary

Prion diseases are transmissible fatal neurodegenerative diseases caused by aberrant metabolism of the cellular prion protein (PrP^C^). The transmissible agent is PrP^Sc^, a misfolded version (conformer) of PrP capable of converting PrP^C^ into PrP^Sc^. PrP^Sc^ can be generated *de novo* in inherited prion diseases due to synthesis of aberrant PrP forms from a mutated PrP gene. Such mutant PrP forms, analogous to other aberrant proteins, should typically be destroyed by various cellular ‘quality control’ (QC) pathways; however, several human diseases result from an eventual breakdown in these QC systems, often due to prolonged bombardment by mutant proteins. We have therefore sought to identify the specific pathways that normally cope with disease-causing misfolded PrPs. By carefully following the generation and turnover of these mutant PrPs in cells, we have discovered an intracellular QC pathway that selectively routes biochemically aberrant PrP species to lysosomes. As the lysosomal system has been implicated as a site for conversion of PrP^C^ to PrP^Sc^, our identification of a mutant-selective trafficking pathway to this compartment may provide a cell biological basis for spontaneous generation of PrP^Sc^ in familial prion disease. Importantly, these findings suggest that eventual changes or breakdown of this QC pathway may contribute to disease progression.

## Introduction

Several diseases are caused by mutations that generate aberrant proteins with adverse cytotoxic consequences [1]. Analysis of the biosynthesis, maturation, processing, trafficking and metabolism of the aberrant protein generated in these diseases is instrumental in identifying the mechanisms underlying pathogenesis. Disease-causing mutations can result in misfolded proteins that are rapidly (and sometimes inappropriately) eliminated by cellular quality control (QC) pathways. In other instances, mutants fail to be efficiently recognized by QC and consequently accumulate excessively. In both situations, the interaction between mutant proteins and QC machinery has emerged as a critical determinant of disease pathogenesis. Therefore, mechanistic analyses of mutant protein metabolism have consistently provided insights into cellular QC pathways whose age-dependent failure is increasingly implicated in various degenerative diseases [2].

For proteins transiting the secretory pathway, the major QC pathways reside in the ER [3]. Considerable insight into ER quality control has come from the analysis of both artificial and naturally occurring mutant proteins. The sum of these studies has led to three general themes. First, multiple QC pathways operate in parallel to recognize different subsets of client proteins. Second, recognition of misfolded proteins is typically mediated by chaperones. Third, the features that distinguish folded from misfolded proteins usually involve exposure of residues or domains (e.g., unpaired cysteines or hydrophobic patches) inappropriate for the environment. While other sites of QC in the secretory pathway have also been proposed, they have been poorly studied [3],[4]. Collectively, these multiple QC systems deal with a remarkably wide range of substrates. Failure to recognize and appropriately triage a mutated protein may underlie many dominantly inherited gain-of-function protein misfolding diseases. Thus, an understanding of the pathogenic mechanisms requires knowledge of the QC pathway(s) that are normally engaged by the aberrant mutant protein.

Prion diseases are one example of disorders in which the generation of aberrant misfolded proteins has dire consequences for the cell. These neurodegenerative diseases can be acquired by a transmissible route, sporadically, or through an inherited mutation. In each case, the central event involves aberrant metabolism of the cell surface prion protein (PrP) [5]. In transmissible prion diseases, a misfolded conformer of PrP (termed PrP^Sc^) directs the templated conversion of normal cellular PrP into additional PrP^Sc^. How or why PrP^Sc^ accumulation leads to neurodegeneration remains largely unknown. A primary obstacle to such studies is the difficulty in classifying cellular alterations as causes, direct consequences or secondary adaptations to PrP^Sc^ accumulation. One way to circumvent this challenge may be to investigate inherited mutations, where the root cause of the disease, a specific mutation in PrP, is established [6]. By determining the effect of the mutation on PrP metabolism, potential events that lead to cellular dysfunction can be identified.

This type of approach has already shed light on the mechanism of pathogenesis of a subset of inherited PrP mutations that occur within the central hydrophobic domain of the protein. These mutations cause increased generation of a transmembrane form of PrP termed ^Ctm^PrP [7]. Elevated ^Ctm^PrP levels can lead to neurodegeneration in both mouse models and humans [7], illuminating one pathway of PrP-mediated cytotoxicity that may even have a broader role in transmissible disease [8]. More generally, these studies led to the discovery that some N-terminal ER translocation signals are intrinsically inefficient [9], may be regulated [10],[11], and could contribute to neurodegeneration [12], illustrating the utility of mutant protein analyses in uncovering broader principles in cell biology.

Interestingly, however, most inherited mutations do not occur within the hydrophobic region, but in the globular C-terminal domain [6]. The mechanism(s) by which such mutations cause disease is not known. These mutations can, in principle, affect many different aspects of PrP metabolism or function. Numerous (and sometimes conflicting) aberrations have been described for individual mutations studied in a wide range of experimental systems [13]–[23]. In some instances, different populations of the same mutant protein are metabolized by different pathways, neither of which is normal [15]. In other cases, different studies examining the same or related mutants come to diametrically opposite conclusions about what is aberrant [16],[24]. Thus, the conclusions from these mutant studies are rather diverse and range from no identifiable effects [25],[26] to nearly quantitative and dramatically abnormal processing [13],[27], sometimes for the same mutant. Which, if any, of these alterations might actually contribute to disease progression versus representing innocuous or even adaptive changes, remains difficult to discern. These observations are further confounded by the fact that only a minor subpopulation of the mutant protein is likely to display altered cellular behavior under normal conditions (given the late-onset of disease), with the major population showing wild type properties. Yet, these minor populations, over long time periods in post-mitotic cells, can nonetheless have significant physiologic consequences in vivo. The challenge therefore is to identify subtle and sometimes minor deviations from normal PrP metabolism that might be a contributing factor in disease.

To address this problem, we have initiated a systematic and quantitative comparison of multiple inherited mutations within the C-terminal globular domain of PrP. Our focus was on identifying the ways that cells distinguish and differentially handle wild type versus mutant PrPs. We reasoned that deviations shared by many or all of the mutants may represent the cellular quality control response to aberrant PrP species. A clear delineation of these pathways is almost certainly of direct relevance to the associated diseases. Not only would such analyses identify the routes of mutant PrP trafficking, but also provide strong candidates for pathways that, when perturbed, would lead to accumulation of aberrant PrP. Indeed, age-dependent decline in QC and degradation pathways is emerging as a common theme in many neurodegenerative diseases, underscoring the importance of defining their role in PrP metabolism. In this study, our analysis has led to the identification of an intracellular trafficking pathway that is shared by several mutant PrPs to selectively route misfolded species for degradation in lysosomes. Interestingly, this misfolded mutant PrP-specific trafficking pathway is dependent not only on the C-terminal mutation but on a highly conserved lysine-based motif in the N-terminus of PrP. These data highlight an unappreciated role for post-ER quality control in PrP metabolism and further suggest important hypotheses for the mechanisms underlying prion disease pathogenesis.

## Results

### Subtle differences in steady state localization patterns of wild type and mutant PrPs

**Figure 1:**
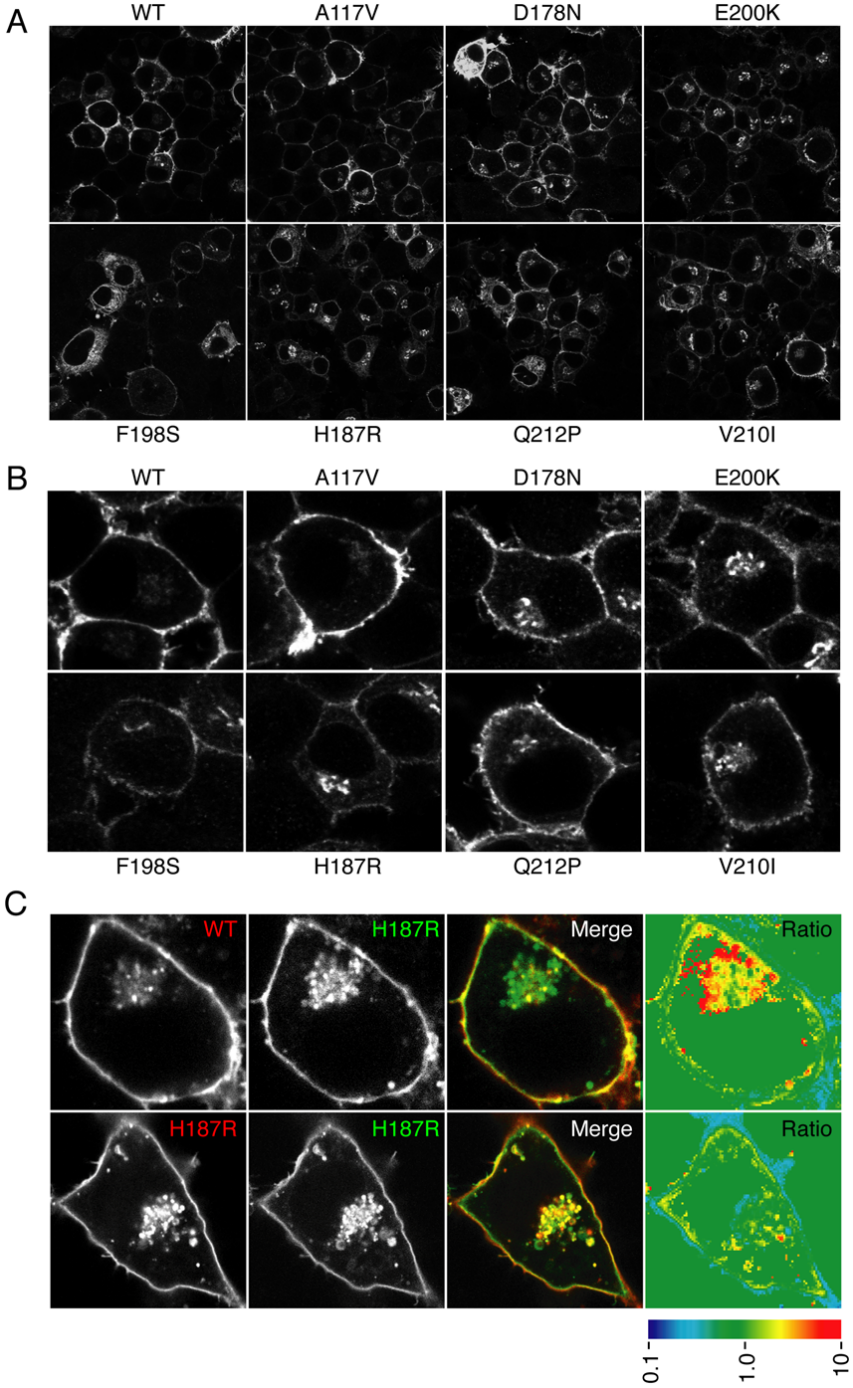
Steady state localization of wtPrP and various disease-causing mutants. (A) Indirect immunofluorescent localization of PrP using the 3F4 antibody in N2a cells transiently transfected with wtPrP or any of 7 different PrP mutants. Identical detector settings were used to image representative fields of cells. (B) Enlarged images of single cells chosen from the corresponding fields from panel A illustrate the overall subtle differences in localization of C-terminal mutant PrPs compared to wtPrP and PrP(A117V). (C) Cells co-expressing fluorescently tagged wtPrP and PrP(H187R) were imaged. Pseudocolored depiction of the mutant∶wt fluorescence ratio in different cellular locales is shown in the last panel (scale is below the image).

Wild type human PrP (wtPrP) and several human disease-causing PrP mutants were expressed in mouse N2a cells and selectively visualized by indirect immunofluorescence with the human-specific 3F4 PrP antibody (Fig. 1A–B). As expected, wtPrP was found predominantly on the cell surface, with varying amounts of an intracellular pool in the ER, Golgi, and endosomal system (Fig. S1A). This pattern is consistent with PrP trafficking through the secretory pathway en route to the cell surface, its constitutive recycling through the endosomal system, and its eventual degradation in lysosomes. This same general pattern was seen in all cells regardless of expression level, although there was some heterogeneity in the relative amounts found in each of the different cellular compartments (Fig. S1A). All PrP mutants also showed localization in the same compartments, including the ER, Golgi, endosomes and the cell surface (Fig. S1B). A panel of representative cells, all at comparable expression levels, illustrates the generally similar patterns of localization for wtPrP and each of the mutants (Fig. 1B).

Among the mutants analyzed, PrP(A117V) was most similar to wtPrP not only in its general pattern of localization, but also in the relative amounts seen in the different cellular compartments. This is consistent with the fact that this mutation is not located within the structured globular C-terminal domain and therefore does not influence the folding of PrP, but is instead known to influence the topology of a small subset (<5%) of total PrP in vivo [7],[28]. Other mutations within the central hydrophobic domain that influence topology [PrP(G114V) and PrP(G131V)] also had no discernable effect on localization (data not shown). By contrast, each of the mutants within the globular C-terminal domain, while displaying the same qualitative pattern of localization, appeared to have a somewhat different relative distribution of PrP among the different compartments. For example, the most obvious case in this experiment is PrP(H187R), where in most cells, the relative intensity of the perinuclear intracellular fluorescence was similar to or greater than that on the cell surface. While cells with this type of distribution were sometimes seen for wtPrP (Fig. S1A), they were much less prevalent. Conversely, cells with nearly exclusive cell surface localization were seen less frequently in PrP(H187R) than wtPrP cells. A similar subtle shift in distribution was also noted for each of the other globular domain mutants, although as with PrP(H187R), this was not a property of every cell. Qualitatively similar effects of these mutants were also observed in HeLa and MDCK cell lines (unpublished observations), indicating that the consequences of these mutations were not species or cell type specific.

Further support for a different relative distribution was provided by analysis of cells co-expressing fluorescently tagged wtPrP and PrP(H187R). Consistent with the analyses of non-tagged proteins, wtPrP-CFP and PrP(H187R)-YFP displayed largely overlapping patterns of localization (Fig. 1C). However, quantitative analysis of their relative ratios in different cellular regions (see the quantitative ratiometric image, Fig. 1C) revealed a clear difference in the peri-nuclear intracellular population. While the ratio of wtPrP-CFP∶PrP(H187R)-YFP was ∼1∶1 at the cell surface, this ratio ranged between ∼2∶1 to ∼10∶1 in the intracellular peri-nuclear regions. The overall ratio within the entire intracellular region was, on average, ∼3∶1. Therefore, both proteins appeared to occupy largely the same cellular locales, albeit with different ratios. These regional differences in ratio could not simply be explained by different fluorescence properties of CFP versus YFP, as co-expression of PrP(H187R)-CFP with PrP(H187R)-YFP showed a much more uniform ratiometric image (Fig. 1C). In addition, exchanging the fluorescent tags on the wt and mutant PrPs still showed increased intracellular fluorescence from the mutant, while co-expression of differently tagged wtPrPs in the same cell showed a relatively uniform ratio of fluorescence throughout (data not shown). Thus, although both wtPrP and PrP(H187R) seem to sample the same cellular regions, they differ in their relative distribution among at least some intracellular compartments.

This difference occurred within the secretory pathway (i.e., after PrP translocation into the ER), as cells blocked in ER-to-Golgi transport with Brefeldin A showed identical, exclusively ER localization patterns for both wtPrP and PrP(H187R) (Fig. S1C). Consistent with this result, in vitro translocation analysis of all of these mutants showed that their import efficiency into the ER was unaffected ([28] and our unpublished results). Only the mutations within the hydrophobic domain [PrP(G114V), PrP(A117V), and PrP(G131V)] showed an effect on translocation, resulting in slightly increased generation of a topological variant, ^Ctm^PrP ([7] and our unpublished results). Together, these observations indicate that the globular domain mutants are qualitatively similar to wtPrP in their ER import, trafficking, and degradation, leading to a very similar pattern of cellular distribution. Nonetheless, differential ratios of the wt and mutant PrPs in certain compartments suggested an effect of the mutations on some aspect of PrP trafficking and/or degradation at a step after translocation into the ER.

### Quantitative single-cell analyses reveal differential mutant PrP localization

**Figure 2:**
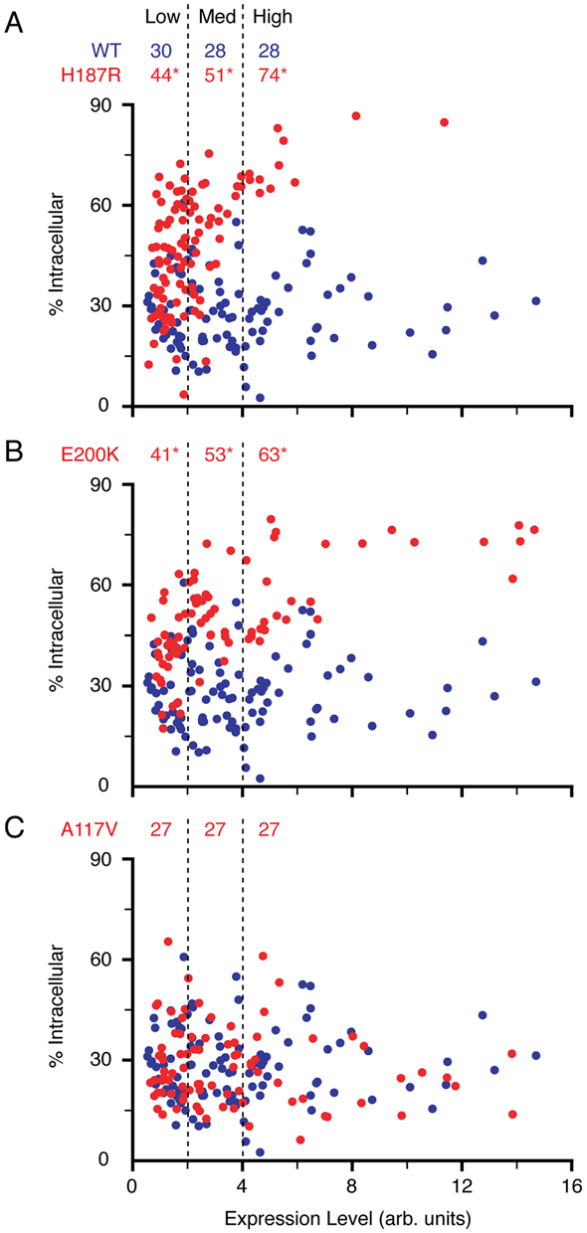
Single-cell quantitative analysis of PrP localization. (A) Cells expressing wtPrP (blue) or PrP(H187R) (red) were immunofluorescently labeled and quantified on a cell-by-cell basis (as detailed in Fig. S2) to determine the percent of total fluorescence found in intracellular compartments. This value (% intracellular) is plotted against expression level, with each point representing an individual cell. Data collected from a single representative experiment is shown. Vertical dashed lines demarcate the boundaries of low, medium and high PrP expression levels that were used to bin cells for statistical analysis. The mean intracellular PrP levels (%) for each of these expression levels is listed for both wtPrP and PrP(H187R). Asterisks indicate statistical significance from wtPrP for points falling within the respective expression levels (p<10^−4^, p<10^−7^, and p<10^−16^ at low, medium, and high expression levels, respectively). (B) Analysis performed as in panel A, but for PrP(E200K). (C) Analysis of PrP(A117V) as in panel A. Note that the entire experiment (panels A–C) was performed at the same time, and that the wtPrP data points are included in each graph for comparison.

The heterogeneity of localization patterns seen with both wtPrP and the mutants, combined with their largely overlapping distributions, precluded clear and direct qualitative comparisons. The co-expression analyses (Fig. 1C), while instructive in detecting a difference between the surface and intracellular populations, were potentially confounded by the presence of fluorescent tags, the possibility of alterations in trafficking due to interactions between wt and mutant PrP, and any differences in the absolute expression levels of wt and mutant PrPs. We therefore turned to quantitative analyses of populations of randomly chosen single cells expressing untagged wtPrP or each of the PrP mutants. Individual cells, chosen randomly and imaged at an arbitrary 1 µm thick confocal section (at mid-nuclear level), were quantified for total, intracellular, and cell surface fluorescence (see Fig. S2). The percentage of total fluorescence (within this confocal section) located intracellularly was then plotted against expression level for each cell (Fig. 2A–C).

When analyzed in this manner, wtPrP showed a wide distribution (ranging from ∼5% to 60%) with an average of ∼28% intracellular PrP (Fig. 2A–C). Surprisingly, this distribution remained almost entirely unaffected by expression level over a very broad range (∼20-fold), suggesting that the normal biosynthetic, trafficking, and degradation pathways for PrP are not easily saturable. PrP(A117V) showed an almost identical distribution, and no statistically significant differences from wtPrP could be discerned (Fig. 2C). By contrast, each of the globular domain mutants displayed several differences from wtPrP [PrP(H187R) and PrP(E200K) are shown in Fig. 2A and 2B]. First, the overall intracellular population was statistically higher [∼56% and ∼52% for PrP(H187R) and PrP(E200K) respectively, relative to ∼28% for wtPrP]. Second, expression-dependent increases in the intracellular population could be observed. When binned into low, medium, and high expressing cells, the PrP mutants showed a statistical difference in the amount of intracellular PrP between the low and high level expressing cells (∼44% versus ∼74% intracellular PrP in cells expressing PrP(H187R) at low or high levels respectively). Importantly however, even among the lowest expressing cells (estimated to be close to physiologic levels of PrP), the mutants remained discernable from wtPrP. Consistent with the qualitative observations, substantial overlap in distribution could be observed between the wtPrP and mutant cells, particularly at the low and medium expression levels.

**Figure 3:**
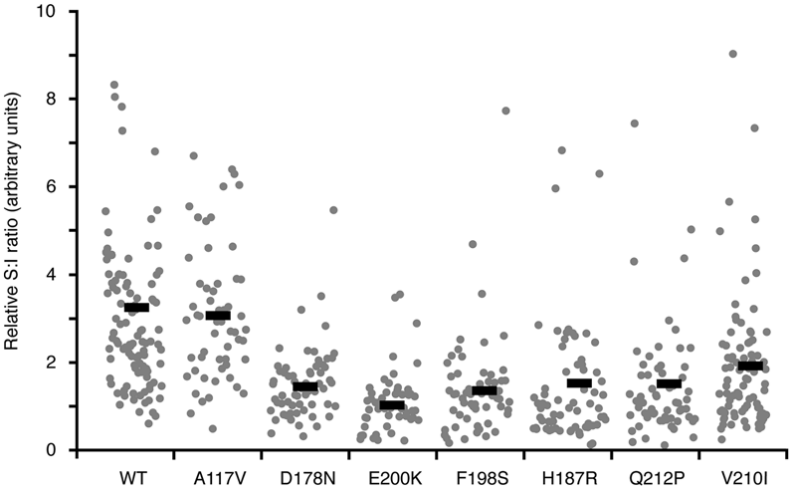
All globular domain PrP mutants display altered localization. Cells expressing wtPrP and the indicated mutants were stained, imaged and analyzed as in Fig. 2. The surface∶intracellular ratio of PrP is plotted for each of the mutants for comparison with wtPrP. Data points represent individual cells from a single representative experiment. All mutants were analyzed together on the same day. Horizontal black bars indicate the mean values for each data set. Each mutant dataset was compared to wtPrP by the Student's t-test and found to be statistically significant in all cases (p<10^−8^), except PrP(A117V).

It is noteworthy that the absolute proportion of intracellular PrP changed among different experiments and appeared to be influenced by culture conditions (e.g., batches of serum, age of cells, relative confluence, and time after transfection). Despite this variability, the relative differences among the mutants remained reproducibly consistent when sufficient numbers of cells were analyzed. Shown in Fig. 3 is the tabulated data from a single experiment comparing wtPrP with seven mutants, showing consistently decreased surface-to-intracellular ratio among each of the globular domain mutants, but not PrP(A117V). The wide heterogeneity among cells, the effect of different culture conditions, the expression level-dependence of localization patterns, and the substantial overlap in distribution between wtPrP and the mutants are likely to explain the previously diverse (and sometimes conflicting) results regarding the effects of PrP mutants. Indeed, many of the previous localization patterns (ER, aggregates, Golgi, etc.) were readily observed in our experiments in subpopulations of cells, emphasizing the importance of more quantitative population studies in identifying overall systematic differences.

### Biochemical identification of a mutant-specific subpopulation of PrP

**Figure 4:**
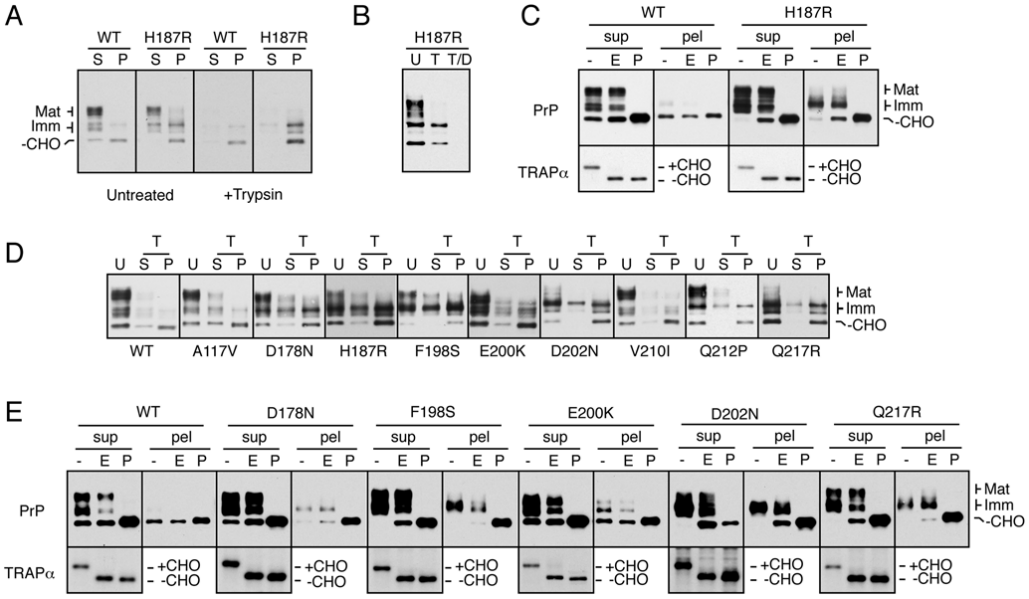
Biochemical identification of a mutant-specific subpopulation of misfolded PrP. (A) Detergent lysates from cells expressing wtPrP or PrP(H187R) were separated into soluble (S) and insoluble (P) fractions, resolved by SDS-PAGE and immunoblotted using the 3F4 antibody. The cells were either analyzed directly (untreated) or first digested with 100 µg/ml of extracellular trypsin to remove cell surface proteins prior to lysis. The migration of different PrP species are indicated on the left: Mat = mature PrP with the full complement of complex glycans; Imm = immature PrP with core glycans; −CHO = unglycosylated PrP. (B) Cells expressing PrP(H187R) were either left untreated (U), digested with 100 µg/ml of extracellular trypsin (T), or digested with trypsin in the presence of 0.2% Triton X-100 detergent (T/D) before harvesting for SDS-PAGE and immunoblotting. (C) Detergent lysates from cells expressing wtPrP or PrP(H187R) were digested with EndoH (E) or with PNGase (P) or left untreated (−), prior to analysis by SDS-PAGE and immunoblotting for PrP. The lower panel shows this blot stripped and re-probed with an antibody against an ER resident glycoprotein, TRAPα. (D) Cells expressing wtPrP or each of 10 PrP mutants were digested with extracellular trypsin, harvested in detergent, separated into soluble (S) and insoluble (P) fractions, and analyzed by immunoblotting. For comparison, one-fourth the amount of total untreated (U) cell lysate is shown. (E) Analysis of the indicated PrP mutants by glycosidase digestions as in panel C.

The differential steady-state distribution of globular domain mutants suggested the presence of a mutant-selective subpopulation that was either trafficked or metabolized differently than wtPrP, resulting in its intracellular localization. To identify and biochemically characterize this population, we analyzed PrP in detergent lysates of N2a cells expressing either wtPrP or the different mutants. In a typical experiment, wtPrP and PrP(H187R) recovered in the detergent soluble fraction (using 0.5% Triton X-100 and 0.5% deoxycholate) showed little or no differences in the glycosylation pattern or isoforms (Fig. 4A; “S” lanes). However, slightly less mutant PrP was consistently observed in the soluble fraction. This detergent soluble wtPrP or PrP(H187R) was quantitatively lost if the intact cells were treated with extracellular trypsin prior to analysis (Fig. 4A). This indicates that the population of wtPrP and mutant PrP on the cell surface share similar solubilization properties and are comparably glycosylated.

By contrast, the detergent insoluble fraction (Fig. 4A; “P” lanes) showed an increase in the PrP isoforms recovered from cells expressing PrP(H187R) versus wtPrP. This insoluble mutant PrP population was intracellular because it was protected from extracellular trypsin added to intact cells (Fig. 4A), but completely digested under the same conditions if cells were permeabilized with detergent (Fig. 4B). The relative amount of this insoluble PrP(H187R) varied based on expression level (consistent with the imaging data in Fig. 2) and culture conditions such that higher expression levels correlated with increased amounts of insoluble, intracellular forms (Fig. S3A and S3B). In some experiments, only a very small proportion of PrP(H187R) was recovered in the insoluble fraction, but in all instances, this was consistently greater than that seen with wtPrP (Fig. S3C). The PrP isoform preferentially enriched in the insoluble fraction of cells expressing PrP(H187R) relative to wtPrP was glycosylated, but not fully modified. By contrast, the unglycosylated isoform of PrP (which likely represents the small proportion that failed to enter the ER [29]) was recovered to a comparable extent for both wtPrP and PrP(H187R). The glycosylated PrP(H187R) in the insoluble fraction was mostly (but not entirely) resistant to digestion with endoglycosidase H (EndoH), but not PNGase (Fig. 4C). This suggests that the majority of detergent-insoluble PrP(H187R) had been processed by Golgi enzymes. Thus, the mutant-specific subpopulation of PrP(H187R), distinguishable by its relative detergent insolubility, is in a post-ER intracellular compartment as had been anticipated by the localization analyses. Its differential glycosylation relative to the major cell surface population further argues for its altered trafficking or processing.

Similar analyses of several other globular domain mutants revealed the existence of an increased population (to varying extents in different experiments) of detergent-insoluble, trypsin inaccessible (intracellular), incompletely glycosylated, EndoH-resistant isoforms (Fig. 4D, 4E, and data not shown). In each case, the increase in this species came at the expense of fully mature, detergent soluble forms when expression levels and film exposure times were carefully matched. By contrast, each of the mutants in the hydrophobic domain did not accumulate PrP species with these characteristics, consistent with their essentially normal localization pattern when analyzed by immunofluorescence. This mutant-specific biochemical species therefore likely corresponds to the differentially localized subpopulation observed in live cells and identified by the single-cell quantitative analyses. Indeed, biochemical features (including relative detergent insolubility) that are shared by multiple prion protein mutants has been previously observed, albeit with a different cell line and different PrP mutants from those in our study [13]. In addition to the EndoH-resistant population of each mutant, a variable amount of detergent-insoluble EndoH-sensitive population was also sometimes observed (Fig. 4C and 4E). This might represent an ER-localized subpopulation of the mutant PrPs that is slightly delayed in its exit to the Golgi [24]. Alternatively, it is possible that glycan trimming on mutant PrPs is either somewhat slower or less efficient than for wild type PrP, leading to an EndoH-sensitive population. Regardless of the explanation, the post-ER EndoH-resistant population is the predominant mutant-specific species observed, consistent with the imaging results in Fig. 1.

**Figure 5:**
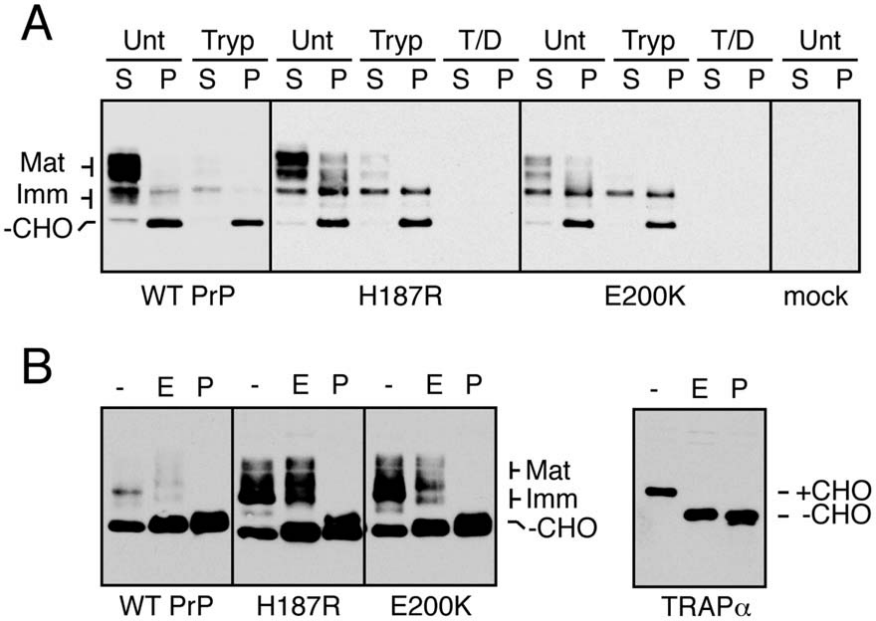
Analysis of PrP mutants in HeLa cells. (A) Wild type PrP, PrP(H187R) and PrP(E200K) were expressed in HeLa cells and analyzed for detergent solubility and surface trypsin digestion as in Figure 4. Mock-transfected cells were also analyzed in parallel. Very long exposures of the blot revealed low level endogenous PrP expression in the mock transfected sample, but insufficient to interfere with analysis of the transfected PrP. (B) Analysis of the detergent-insoluble fraction of either WT or mutant PrPs for glycosidase digestions. TRAPα in the detergent soluble fraction is shown as a control for the digestions.

Several of the mutants were also analyzed in HeLa cells, a completely unrelated human cell line that expresses very low (but detectable) levels of endogenous PrP. As in N2a cells, PrP(H187R) and PrP(E200K) expressed in HeLa cells showed increased amounts of detergent-insoluble, incompletely glycosylated, intracellular forms (Fig. 5A). This increase was typically accompanied by a reduction of the fully mature detergent-soluble species, and was especially evident for some mutants (e.g., E200K). The detergent-insoluble mutant-specific population was mostly (but not entirely) resistant to deglycosylation with EndoH (Fig. 5B), indicating its primarily post-ER localization. Thus, unlike the highly species-specific nature of prion-templated conversion from PrP^C^ to PrP^Sc^, the folding, trafficking, and metabolism of PrP mutants appears to be more generally conserved. This is consistent with intracellular protein trafficking and metabolism being mediated by very generic and highly conserved machinery.

Furthermore, the finding of similar results in two cell lines that express different amounts and species (human versus mouse) of endogenous PrP suggests that the behavior of PrP mutants may not be significantly influenced by the co-expressed wild type counterpart. Indeed, in the short term of our experiments, the presence or absence of endogenous PrP in co-expression experiments (e.g., as in Fig. 1C) did not seem to influence mutant PrP localization. Whether the converse effect occurs (of mutant PrP altering wild type PrP properties) is unclear from our studies, but seems plausible given that PrP can interact with itself. Earlier observations suggest such an effect [23], and would be consistent with the dominant nature of disease inheritance.

### The mutant-specific subpopulation of PrP is misfolded

**Figure 6:**
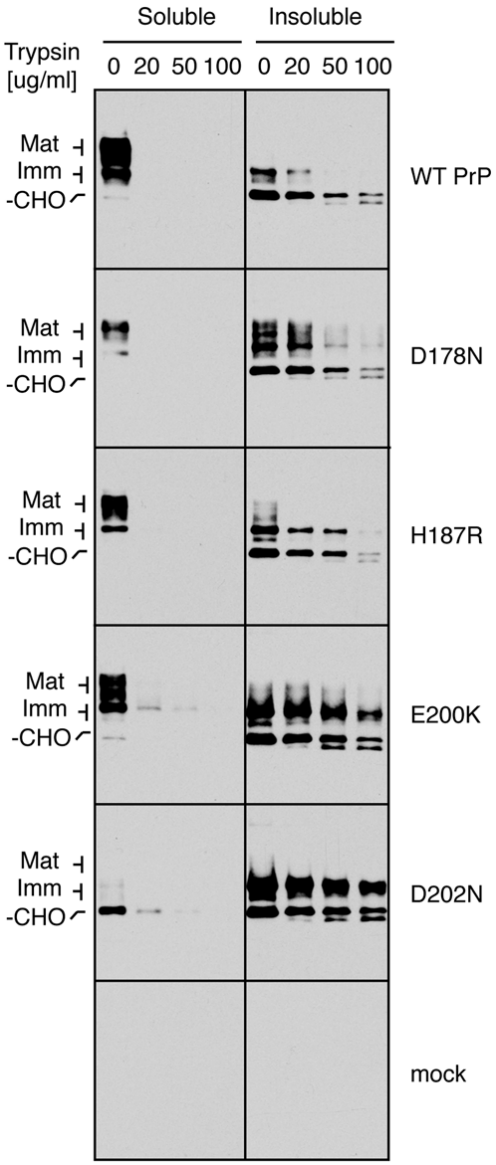
Limited protease digestion analysis for folding status of PrP mutants. Total detergent lysates of the indicated PrP constructs were digested with various concentrations of trypsin on ice before separation into soluble and insoluble fractions that were analyzed by immunoblots. Note that these digestion conditions are significantly milder than that used for analysis of surface exposure (e.g., in Fig. 4A and 5A), where trypsin fully digests the PrP mutants.

Altered localization, detergent-insolubility, and incomplete glycan modification are all indirect indicators of protein misfolding. To directly assess whether the specific subpopulation of mutant PrPs that display these characteristics are indeed folded differently, we employed limited trypsin digestion (Fig. 6). In this experiment, total detergent lysates from PrP-expressing cells were treated on ice with increasing concentrations of trypsin, followed by separation into soluble and insoluble fractions. As expected, essentially all mature wild type PrP was completely in the supernatant fraction, and this population was highly sensitive to trypsin. By contrast, unglycosylated PrP (presumably a cytosolic form that is folded differently) was quantitatively in the insoluble fraction and showed notably more trypsin resistance. A small amount of immature glycosylated PrP was also seen in the insoluble fraction and displayed modest trypsin resistance. The differences in trypsin sensitivity among the different PrP forms illustrates the utility of this assay in discriminating among them.

When mutant PrPs were analyzed by this assay, each one displayed clear differences from the wild type (Fig. 6). Most notably, a higher proportion of the mutant PrPs were found in the insoluble fraction as incompletely glycosylated forms (as characterized in Fig. 4 and 5). This species in each mutant was preferentially resistant to trypsin compared to the highly sensitive fully glycosylated soluble species from the same mutant. The unglycosylated species of each mutant showed comparable resistance to the unglycosylated species of wild type PrP, suggesting that this form is similarly folded regardless of the mutation. We can conclude from this analysis that by the measure of limited protease accessibility, the immaturely glycosylated and detergent-insoluble species that is enriched in each mutant is folded differently than the soluble fully mature species of either wild type PrP or the mutants.

It is worth emphasizing that detergent insolubility and trypsin resistance should not be taken to necessarily indicate aggregation. Rather, these are simply convenient assays that allow enrichment of this species of PrP which, due to its altered folding by as yet undefined ways, causes a change in its biochemical properties. Similar differences in detergent solubility have been described before for some PrP mutants [13],[14],[21],[23] and could represent residence in different membrane environments. Indeed, as described in subsequent sections, the efficient ER exit, accessibility to glycosidases, and the absence of punctate structures by imaging all argue against a grossly aggregated or insoluble species in vivo.

### Spatio-temporal analysis of mutant PrP trafficking

**Figure 7:**
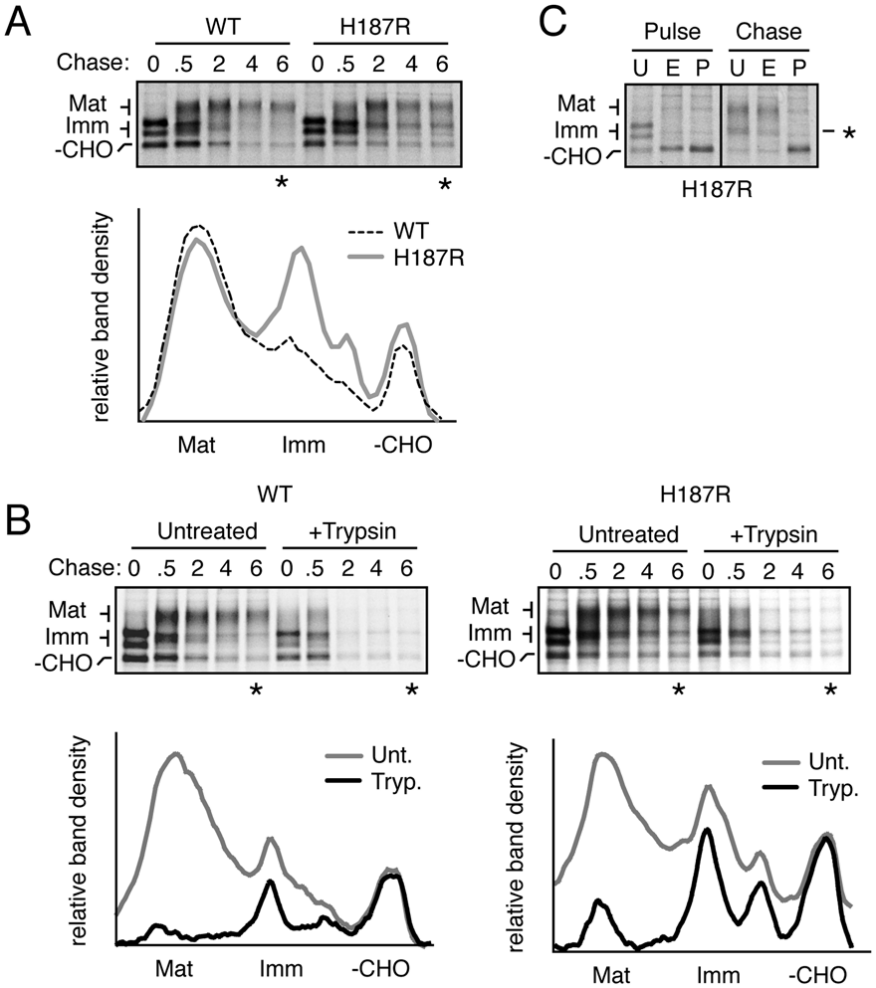
Immature mutant PrP species persist in post-ER intracellular compartments. (A) Pulse-chase analysis of cells expressing wtPrP and PrP(H187R). Cells were pulsed for 10 minutes with ^35^S-methionine, chased for the times indicated (in hours) and total cell lysates were immunoprecipitated for PrP. The asterisk (*) indicates lanes whose desitometric profile is shown below. (B) Cells expressing wtPrP or PrP(H187R) were analyzed by pulse-chase analysis as described in panel A. Just prior to harvesting the cells, they were either treated with 100 µg/ml of extracellular trypsin (+Trypsin) or were left untreated. The asterisk (*) indicates lanes whose densitometric profile is shown below. (C) Pulse (0 hours) and chase (0.5 hours) samples from PrP(H187R) expressing cells were digested with EndoH (E), PNGase (P) or left untreated (−). Note that essentially all of the PrP is converted from EndoH sensitive (at pulse) to resistant forms (at chase), including the immature forms (indicated by asterisk).

To determine the origin of the mutant-specific intracellular subpopulation of PrP, we turned to pulse-chase analyses. Upon pulse labeling transfected cells for 10 minutes with ^35^S-methionine, wtPrP is synthesized in three forms corresponding to unglycosylated (∼25%), singly-glycosylated (∼25%), and doubly-glycosylated (∼50%) PrP (Fig. 7A). Upon chase for 30 minutes, the glycosylated forms were trimmed to a slightly smaller molecular weight species (consistent with mannosidase action in the cis-Golgi), and matured to higher molecular weight complex glycosylated species, consistent with transit through the Golgi stacks (Fig. 7A, compare lanes “0” and “0.5”). The fully mature (Golgi and post-Golgi) form represents ∼40% of the total cellular PrP at 30 minutes and is the predominant (>60%) form seen after 2 hours of chase. About half of this mature form was then degraded over the course of the next 4 hours, consistent with an approximate 6 hour half life of cell surface PrP observed in previous studies [30].

PrP(H187R) looked qualitatively similar to wtPrP at early time points, as no differences were noted in its core glycosylation and initial trimming up to 30 minutes of chase (Fig. 7A). Mature PrP(H187R) was also generated by 30 minutes, reaching a maximal amount by 2 hours and decaying with similar kinetics to wtPrP. The principal difference from wtPrP was that a small proportion of the trimmed, immature form of PrP(H187R) generated at 30 minutes persisted throughout the chase period (Fig. 7A). This band decayed with kinetics slower than that of the mature form, leading to a progressive increase in its relative prominence. By the 6 hour chase point, this immature species represented ∼30–50% of total PrP(H187R), whereas it was largely absent from wtPrP (see densitometry analysis, Fig. 7A). Analyses of other globular domain mutants such as PrP(E200K) and PrP(D178N) showed very similar results, while by contrast, PrP(A117V) mirrored wtPrP and showed no persistence of an immature species (Fig. S4A, S4B, and data not shown).

Thus, a normally transient, glycan-trimmed minor population of immature PrP seen during the normal maturation of wtPrP persists for a prolonged period during the biogenesis of several globular domain mutants, but not a hydrophobic domain mutant. The migration of this immature population corresponds to the migration of the insoluble, intracellular, EndoH-resistant, mutant-specific form characterized at steady state (Fig. 4). It is important to note that while this persistent immature mutant-specific PrP species represents a very small proportion of the total PrP synthesized (∼10–20%), its relatively long half-life compared to the other PrP forms explains how this species can nonetheless represent a sufficient population of total PrP at steady state to allow detection by both immunoblotting and immunofluorescence.

We next addressed the location and nature of this minor subpopulation of PrP over time by combining pulse-chase analysis with assays of subcellular localization. Extracellular trypsin digestion at each time point during the pulse-chase showed that the mutant-specific immature species, but not the mature species, was largely shielded from the cell surface (Fig. 7B). Densitometric analysis of the 6 h chase point shows that the mature species was nearly fully digested, while the immature species were mostly shielded. As expected, the unglycosylated species was completely protected due to its cytosolic localization. Glycosidase digestion showed that the immature species becomes resistant to EndoH by 30 minutes of chase, concomitant with the observed glycan trimming that likely represents the action of cis-Golgi mannosidases (Fig. 7C). This indicates that the mutant-specific species has exited the ER (consistent with the analyses of PrP at steady state), but does not acquire mature glycans or reach the cell surface efficiently.

### Post-ER metabolism of misfolded mutant PrP

The rapid acquisition of EndoH resistance of the mutant-specific immature species indicates that it passes the major chaperone-based quality control systems in the ER without significant impediment. This could be explained in two ways. One possibility is that this species folds correctly when in the ER environment (and hence, does not become a substrate of the QC machinery), but misfolds upon delivery to the different environment of the Golgi. Alternatively, it might misfold in a manner that is invisible to the ER QC systems, allowing its exit. In order to distinguish between these two models, we combined pulse-chase analyses with assays of detergent insolubility.

**Figure 8:**
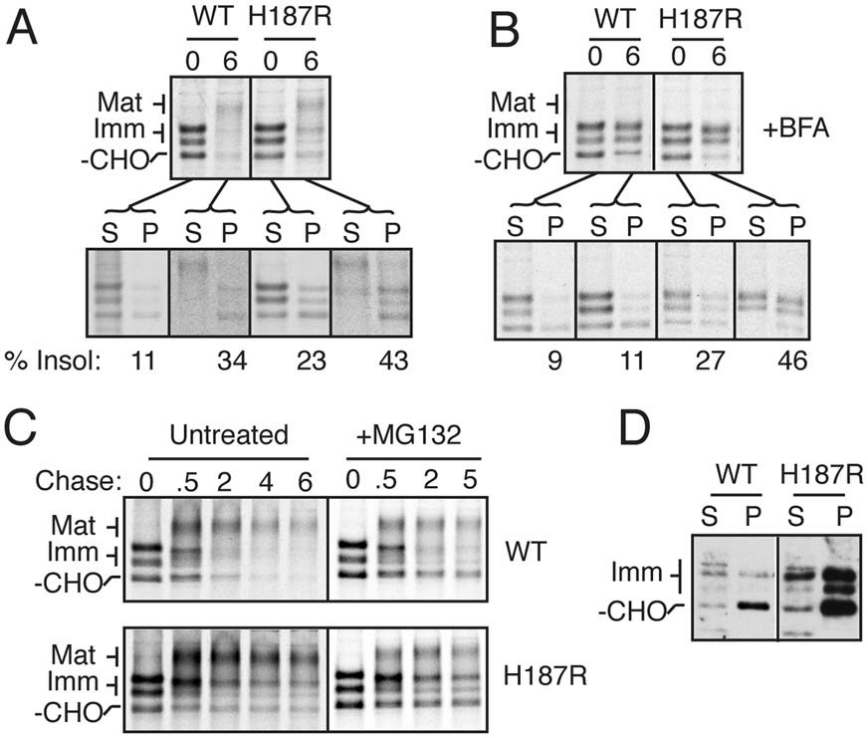
PrP mutants pass ER quality control and are not degraded by proteasomes. (A) Pulse-chase samples from wtPrP and PrP(H187R) expressing cells were separated into detergent soluble (S) and insoluble (P) fractions, immunoprecipitated, and analyzed by autoradiography. (B) Pulse-chase analysis of wtPrP or PrP(H187R) expressing cells as in panel A, but in the presence of 10 µg/ml of Brefeldin A, an inhibitor of ER to Golgi trafficking. (C) Pulse-chase analyses of wtPrP and PrP(H187R) in the absence or presence of 5 µM MG132, a proteasomal inhibitor. (D) A4 cells expressing wtPrP or PrP(H187R) were separated into detergent soluble (S) and insoluble (P) fractions and analyzed by immunoblotting for PrP.

Upon pulse labeling, when PrP is quantitatively in the ER (i.e., EndoH sensitive with untrimmed glycans), we could detect a difference in relative solubility between wtPrP and PrP(H187R) (Fig. 8A). As expected, PrP(H187R) displayed a larger proportion of detergent insoluble PrP isoforms at the earliest point of PrP biogenesis (∼23%, compared to ∼11% for wtPrP). This insoluble mutant PrP population persisted during the 6-hour chase, while the soluble population was converted into mature species. Similar results were obtained for PrP(E200K) (data not shown). We therefore conclude that the globular domain mutants of PrP display altered folding immediately upon their synthesis and entry into the ER. However, this subpopulation is neither recognized by ER quality control, nor retained in the ER. Instead, it is fully competent for ER exit and reaches the Golgi, where it is accessible to cis-Golgi glycosidases. However, it fails to mature further, retains its altered folding status, and does not reach the cell surface.

Additional evidence to support the notion that this mutant-specific species was not recognized by ER-based QC was provided by the failure of this subpopulation to be degraded even upon prolonged retention in the ER with Brefeldin A (Fig. 8B). Furthermore, this species was not significantly stabilized upon proteasome inhibition, the degradation pathway utilized by ER-based QC (Fig. 8C). Remarkably however, this detergent insoluble population of globular domain mutants is not intrinsically refractory to ER-associated degradation (ERAD). In cells deficient in GPI anchor addition (A4 cells), mutant PrPs become detergent insoluble as in normal cells (Fig. 8D), yet are degraded quantitatively from the ER by a proteasome-dependent retrotranslocation pathway [31]. Thus, this detergent-insoluble subpopulation of mutant PrPs is competent for retrotranslocation and degradation; however, in normal cells, it is apparently misfolded in a manner that makes it invisible to the ER-based QC pathways.

### Misfolded PrP mutants are degraded in the endo-lysosomal system

**Figure 9:**
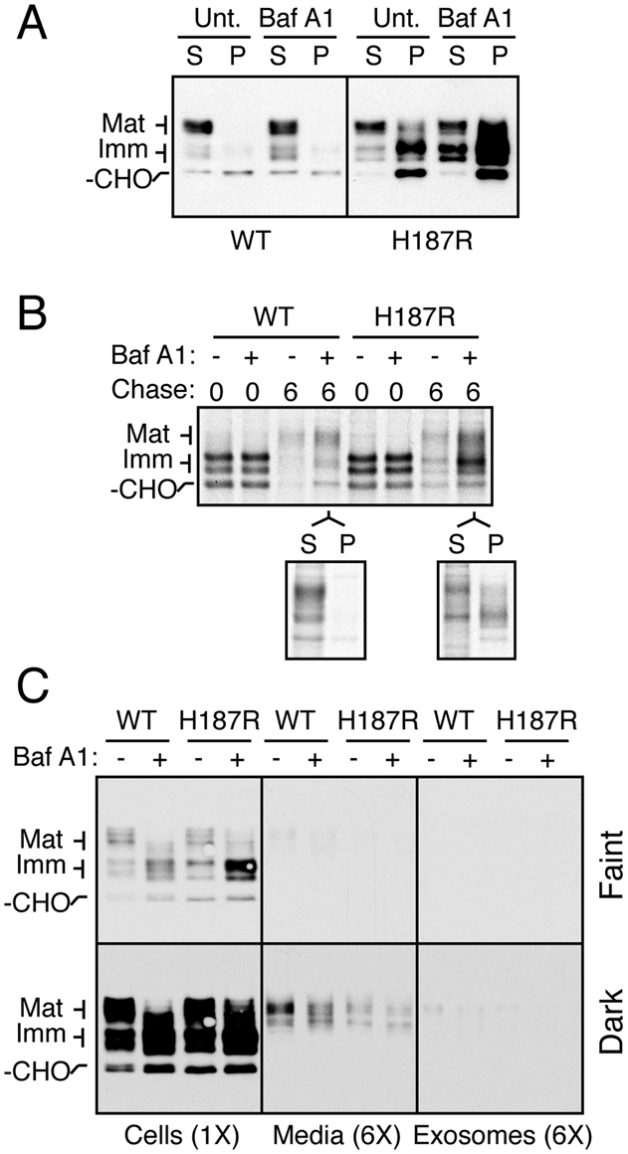
PrP mutants are metabolized in acidic intracellular compartments. (A) N2a cells expressing wtPrP or PrP(H187R) were treated with 0.1 µg/ml of Bafilomycin A1 or vehicle for 6 hours before analyzing total cell lysates by immunoblotting. (B) Pulse-chase analysis of cells expressing wtPrP or PrP(H187R) in the absence (−) or presence (+) of 0.1 µg/ml of Bafilomycin A1. Indicated chase samples were further fractionated into detergent soluble (S) and insoluble (P) components. (C) Cells expressing wtPrP or PrP(H187R) were treated with Bafilomycin A1 or vehicle for 12 hours before analyzing total cell lysates, the conditioned media, and exosomes by immunoblotting. Six-fold relative amount of the media and exosome samples were analyzed. Two exposures of the blot are shown.

Despite exit from the ER, the immature mutant-specific population of PrP does not reach the cell surface. Instead, it appears to be slowly lost from cells by a proteasome-independent pathway. Since the major non-proteasomal site of protein degradation in the secretory pathway is in the lysosome, we evaluated a role for acidic compartments in mutant PrP metabolism. Perturbation of endo-lysosomal function with Bafilomycin A1 (an inhibitor of the vacuolar type H^+^- ATPase) led to the steady-state enhancement of the mutant-specific detergent-insoluble form of PrP(H187R) (Fig. 9A), suggesting the involvement of acidic compartments in its turnover. This conclusion was further confirmed by pulse-chase experiments, where we observed that the immature species of mutant PrP was completely stabilized in the presence of Bafilomycin A1 (Fig. 9B). Mature species of both wtPrP and PrP(H187R) were also stabilized, as expected from previous studies showing degradation of surface PrP by the endo-lysosomal system. Thus, the loss of misfolded mutant PrP(H187R) relies on acidic compartments, indicating that this species is either degraded in lysosomes or excreted from the cell (e.g., on exosomes or via secretory lysosomes).

To investigate how mutant PrP was being metabolized, we analyzed both total protein and exosomes from the conditioned media of PrP-expressing cells (Fig. 9C). A small but detectable amount of mutant and wild type PrP were found in the media and in exosomes from N2a cells. However, the amount recovered from PrP(H187R) media was both slightly less than for wild type PrP, and far too low to explain PrP(H187R) loss by excretion. Indeed, upon Bafilomycin A1 treatment, the immature species of PrP(H187R) accumulated inside cells to far higher levels than seen in the media in the absence of Bafilomycin A1. This means that essentially none of the PrP forms sensitive to Bafilomycin A1 inhibition are being lost via excretion from the cell. Thus, the immature species of mutant PrP is metabolized intracellularly in a manner dependent on acidic compartments. Consistent with this conclusion, chloroquine (another lysosome alkalinizing agent) also stabilized the glycosylated forms of PrP(H187R) (data not shown). When considered together with the lack of proteasome involvement (Fig. 8C), these results are consistent with a model in which the subpopulation of misfolded mutant PrPs pass quality control inspection at the ER but are segregated in a post-ER compartment and routed to lysosomes for degradation.

### A role for the N-terminus in mutant PrP metabolism

Interestingly, although the immature species of mutant PrPs are trafficked to and degraded in lysosomes, this subpopulation neither accumulates in this compartment nor colocalizes significantly with lysosomal markers (such as Lgp-120) or lysotracker (data not shown). This indicates that upon reaching the lysosome, its degradation is probably relatively rapid (evidenced by its dramatic stabilization upon Bafilomycin 1A treatment). Our data further suggests that this mutant subpopulation traffics from the ER to Golgi in under 30 minutes (Fig. 7A). Yet its overall half-life appears to be at least as long as the mature form (∼6 hours), suggesting that in its trafficking from ER-Golgi-lysosome, the rate limiting step is likely to be delivery from the Golgi to lysosome. Consistent with this interpretation, the differentially localized mutant PrP is preferentially enriched in a perinuclear region, which in earlier studies was shown to co-localize with both Golgi and endosome markers [29]. Both of these compartments are major centers of protein sorting for a wide variety of secretory and membrane proteins. Sorting is typically mediated via sorting motifs that are recognized by their respective receptors or adaptors.

**Figure 10:**
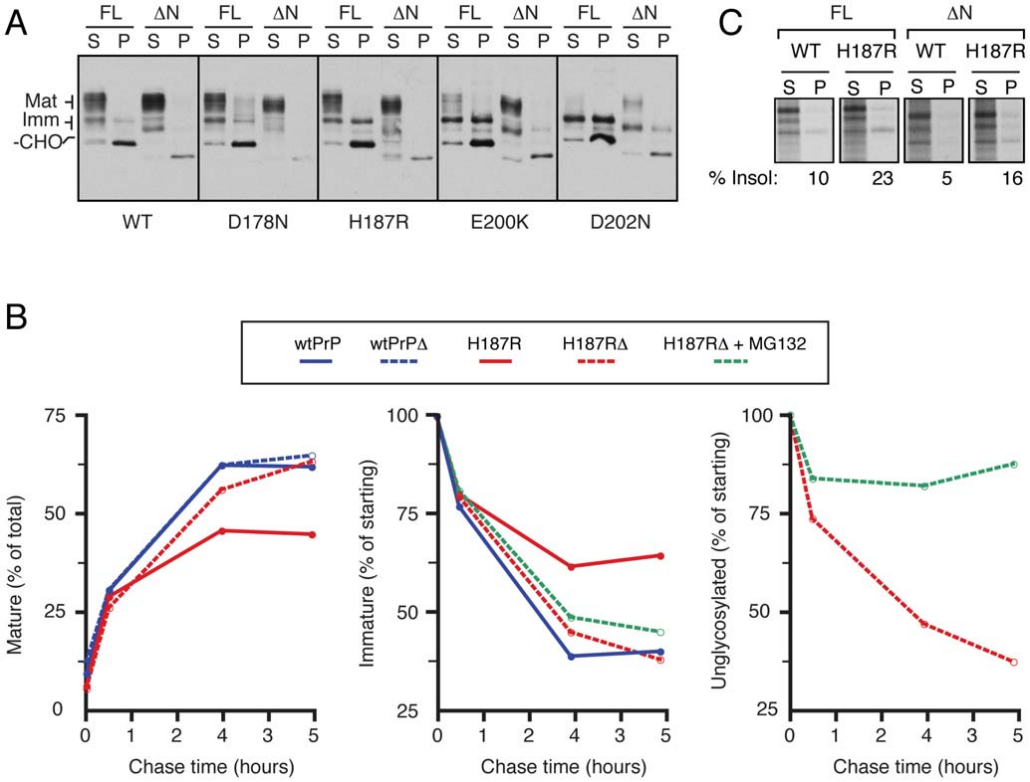
The N-terminus of PrP modulates mutant PrP metabolism. (A) The full length (FL) or N-terminally deleted (ΔN, lacking residues 23–48) constructs for wild type and mutant PrPs were analyzed by the detergent solubility assay. In each case, deletion of the N-terminus resulted in decreased insoluble forms. A corresponding increase in the fully mature soluble form is apparent in most cases. (B) Pulse chase analyses (as in Fig. 7A) of wtPrP, PrP(H187R), wtPrPΔN and PrP(H187R)ΔN were quantified by phosphorimaging. The left panel plots the appearance of fully mature species (as a proportion of total PrP) over time. The middle panel shows the time course of disappearance for immature glycosylated species (plotted as a percent of the amount present at pulse). The inclusion of 5 µM MG132, a proteasome inhibitor, had no effect on the immature species for PrP(H187R)ΔN. The right panel shows the fate of unglycosylated PrP, without or with 5 µM MG132. (C) Lysates harvested after pulse labeling with S^35^-methionine from cells expressing wtPrP, wtPrPΔN, PrP(H187R) and PrP(H187R)ΔN were separated into detergent soluble (S) and insoluble (P) fractions, immunoprecipitated, and analyzed by autoradiography. The percent of labeled PrP that is insoluble is indicated below the respective panels.

To gain insight into the misfolding-selective sorting operating on mutant PrP, we sought to identify potential sorting determinant(s) by analyzing PrP deletion mutants. We found that deletion of residues 23–48 (the ΔN constructs) markedly reduced the amount of detergent-insoluble, immaturely glycosylated species of several PrP mutants relative to their full length (FL) counterparts (Fig. 10A). In most cases, this decrease was accompanied by a relative increase in the fully mature, detergent-soluble species (Fig. 10A). In all but the most severe mutant [e.g., PrP(D202N)], deleting the N-terminal domain normalized the behavior in the solubility assay to near wild type levels. Even for PrP(D202N), a significant degree of normalization was observed. A predominantly cell surface localization of the fully mature species for the ΔN constructs was confirmed by both trypsin accessibility assays and indirect immunofluorescence (data not shown).

Analysis of PrP(H187R) and PrP(H187R)ΔN by pulse-chase showed that the ΔN construct generated mature PrP species in amounts and kinetics very similar to wild type PrP (Fig. 10B; left graph). The improved maturation efficiency of PrP(H187R)ΔN was accompanied by a concomitant decrease in immature PrP species (Fig. 10B; middle graph). This effect was not a consequence of selective ER degradation of immature species of PrP(H187R)ΔN as it was not stabilized by MG132 (Fig. 10B; middle graph). As expected, the non-translocated PrP species in the same samples were indeed stabilized by MG132, thereby serving as a useful internal control (Fig. 10B; right graph). Thus, by both steady state and pulse-chase analyses, deletion of the N-terminal domain significantly modulates the maturation of several PrP mutants, but has little or no effect on wtPrP.

Enhanced maturation of PrP(H187R)ΔN could transpire because the N-terminal deletion either prevents the initial misfolding of the C-terminal globular domain or prevents its selective retention within the secretory pathway. At present, we cannot fully discriminate between these possibilities. However, we did observe that the detergent insolubility of pulse-labeled PrP(H187R) was only partially improved by the N-terminal deletion (Fig. 10C), even though subsequent maturation of PrP(H187R)ΔN was essentially indistinguishable from wild type PrP (Fig. 10A, 10B). Thus, improved generation of fully mature species of PrP(H187R)ΔN cannot fully be explained by an effect only on its initial folding in the ER. This may suggest that the N-terminus could also have a role in modulating the intracellular trafficking of mutant PrP though the secretory pathway, although this remains to be investigated. Thus, two elements, a misfolded C-terminal domain and the extreme N-terminus, collude to influence the metabolism of PrP mutants.

**Figure 11:**
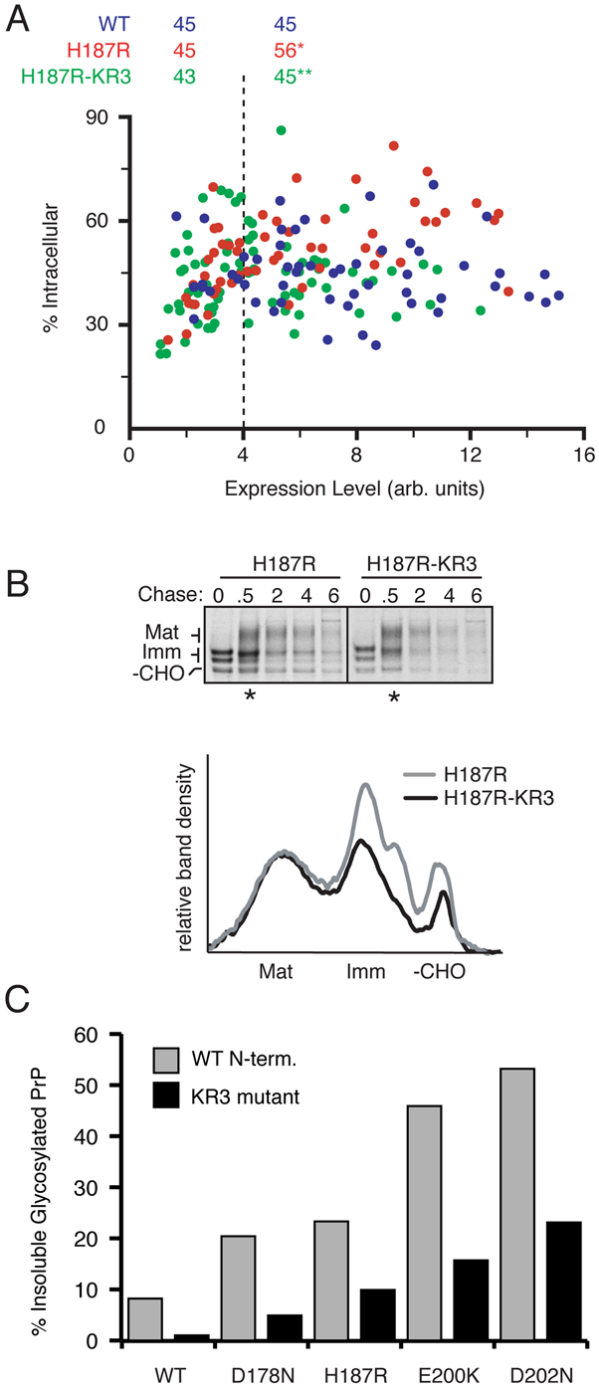
Conserved Lysines in the N-terminus modulate the fate of PrP mutants. (A) Single-cell quantification of localization as in Fig. 2 was performed on cells expressing wtPrP (blue) , PrP(H187R) (red) and PrP(H187R)-KR3 (green), construct in which three conserved Lysines in the N-terminus were changed to Arginines. PrP(H187R) was statistically different from wtPrP (*, p = .003), and PrP(H187R)-KR3 was statistically different from PrP(H187R) (**, p = .0006). (B) Pulse-chase analysis of cells expressing PrP(H187R) and PrP(H187R)-KR3, performed as in Fig. 7A. The asterisks indicate lanes whose desitometric profile is shown below. (C) Cells expressing the indicated constructs either lacking or containing the KR3 mutation in the N-terminus were analyzed by the detergent solubility assays as in Fig. 10A. The percent of each construct in the detergent-insoluble pellet was quantified and plotted.

In order to narrow the motif within this N-terminal domain that influences the generation and/or metabolism of misfolded PrP, we engineered a version of PrP(H187R) in which 3 highly conserved lysine residues (at positions 23, 24 and 27) were changed to arginines [PrP(H187R)-KR3]. This construct was then quantitatively assessed for localization using our single cell analyses (Fig. 11A). Remarkably, this conservative mutation led to a decrease in the amount of intracellular PrP such that the relative cellular distribution of PrP(H187R)-KR3 resembled that of wtPrP, while being statistically different from that of PrP(H187R) (p = 0.0006). Biochemical analyses of PrP(H187R)-KR3 revealed the basis of this effect. We found that in pulse-chase experiments, a higher proportion of PrP(H187R)-KR3 was converted into mature forms at the expense of immature species that normally predominate during the biogenesis of PrP(H187R) (Fig. 11B). This is most readily seen at the 30 minute chase point (see densitometry in Fig. 11B), where the ratio of mature to immature species is significantly increased for PrP(H187R)-KR3. Analysis of total glycosylated PrP species at steady state for detergent solubility showed that the KR3 mutation reduced the percent of insoluble species for each of several mutants, albeit to somewhat different levels, and to a lesser degree than the ΔN deletions (Fig. 11C). Thus, the N-terminus, and in particular a lysine-based motif, appears to be involved in the fate of several mutant PrPs by influencing either their initial biosynthesis and/or their subsequent intracellular trafficking.

## Discussion

We have quantitatively analyzed the trafficking and metabolism of disease-causing PrP mutants. Our aims were two-fold. First, we hoped to gain insight into the QC pathways that discriminate wtPrP from mutants that are ostensibly prone to misfolding. Second, we wanted to determine whether many or all of the PrP mutants would share certain features in their metabolism that could represent potential pathways of direct importance to disease pathogenesis. By determining how cells metabolize PrP mutants relative to the wild type counterpart, we discovered a prominent role for post-ER pathways of differential sorting and trafficking. Interestingly, we found that despite the multitude and diversity of ER quality control pathways, all of the PrP mutants we analyzed were refractory to recognition by these components. They are instead recognized in a more distal compartment of the secretory pathway and sorted for degradation in lysosomes. The implications of these findings for inherited and transmissible prion disease as well as cellular quality control in general are discussed in turn below.

### Implications for pathogenesis of prion diseases

Our primary conclusion that disease-causing PrP mutations use a QC pathway that culminates in lysosomal degradation has several potential implications for inherited prion disease pathogenesis. The most important is that reduced lysosomal function would lead to accumulation of misfolded PrP species. This could represent a mechanism of neuronal cytotoxicity in inherited prion disease. Indeed, age-dependent changes in the lysosomal system such as decreased activity of lysosomal hydrolases and decreased regulation of lysosomal pH have been documented [32]. Such alterations have been proposed to contribute to the slow and age-dependent progression of various protein misfolding disorders including Alzheimer's disease and Parkinson's disease [33]–[35], and could also contribute to the pathogenesis of inherited prion diseases.

In addition, increased production of misfolded PrP species may lead to saturation of the trafficking pathway from the trans-Golgi network (TGN) to lysosomes. It is unlikely that this pathway is used solely by PrP as most cellular trafficking and QC systems have a wide range of substrates. This means that increased traffic of misfolded PrPs (as would occur with these mutants) could lead to compromised function of the secretory pathway, saturation of the Golgi QC machinery, and/or inappropriate secretion of immature or misfolded cellular proteins. In addition, sorting of lysosomal enzymes that are critical for the degradative capacity of this compartment could be compromised. This would lead to reduced lysosomal activity, perhaps in an age-dependent manner [32], thereby causing increased accumulation of aberrant PrP species (and other cellular substrates) within the lysosome. Undegraded misfolded PrPs in the lysosome may further exacerbate lysosomal dysfunction. Indeed decreased function of lysosomal cysteine proteases has been shown to be associated with misfolded PrP accumulation in prion disease [36],[37]. Additionally, scrapie infected neurons have been shown to accumulate large numbers of lysosomes containing PrP in regions of spongiform change in the brain [38]. Thus, lysosomal dysfunction could be a shared contributing event in both familial and transmissible prion diseases.

Lysosomal trafficking could also play a role in the spontaneous conversion of some mutant PrPs into the PrP^Sc^ isoform. While not all inherited PrP mutants generate infectivity (e.g., A117V), a few mutants (e.g., E200K or D178N) are unambiguously converted to PrP^Sc^. Lysosomes (or related acidic compartments) have long been implicated as the site of conversion of PrP^C^ to PrP^Sc^ during transmissible prion disease [30],[39],[40], suggesting that this environment may be especially conducive to PrP misfolding or re-folding. It is therefore plausible that routing of misfolded mutant PrPs to acidic compartments when combined with decreased lysosomal degradation (as might occur during aging), may favor its spontaneous conversion to PrP^Sc^.

### Quality control in the Golgi versus the ER

Our data indicate that wild type and mutant PrPs are distinguished and trafficked differentially within the cell after they have left the ER, the predominant site of QC in the secretory pathway. This conclusion is consistent with several previous observations providing evidence for altered mutant PrP species in post-ER intracellular compartments on the basis of imaging, glycosylation analysis, or inhibitor studies [14],[27],[41],[42]. However, many studies have implicated ER-based QC and degradation as the major route for mutant PrP metabolism. These latter studies have largely relied on two types of observations, each of which merits closer scrutiny. First, mutant PrPs have typically been observed to be ER-localized (at least partially retained), suggesting engagement of ER QC pathways [15],[17],[27]. In some instances, interactions with chaperones have also been observed [15]. What has been unclear in such studies is how quantitative or uniform the retention is, and whether it is dependent on overexpression. In some instances, viral-based expression at very high levels may explain the observations. Indeed, we too observe some cells in our experiments that show ER localization (Fig. S1), often in the highest expressing cells. However, this was not consistent across all expression levels, in contrast to the post-ER population we describe here.

The second line of evidence for ER-based QC is the accumulation of unglycosylated PrP upon prolonged proteasome inhibition [16],[43],[44]. However, PrP translocation was later found to be partially inefficient [9],[24],[29], indicating that at least some unglycosylated PrP is generated from non-translocated material. In addition, translocation of PrP is decreased even further during ER stress [10],[45], which is a secondary consequence of proteasome inhibition. More importantly, when translocation efficiency is improved, the proteasome inhibitor-dependent accumulation of unglycosylated PrP could be completely prevented [10],[29]. This argues strongly for unglycosylated cytosolic species being generated primarily, if not exclusively from inefficient forward translocation and not ER-based QC. Furthermore, a precursor-product relationship between ER lumenal PrP and cytosolic PrP has never been shown formally by pulse-chase studies in cases where ER-based QC of PrP mutants has been proposed. This is in contrast to our direct demonstration of ER-based QC and retrotranslocation of PrP in GPI-anchoring deficient cells [31]. We therefore believe that we would have detected ER-associated degradation of mutant PrPs by these same assays if it were occurring to any appreciable degree. Instead, we favor a model of mutant PrP QC involving post-ER compartments of the secretory pathway, potentially the Golgi. It is interesting to note that different forms of PrP use distinct pathways for their disposal, ranging from ERAD, cytosolic QC, and lysosomal degradation. Among these, the post-ER pathway described here seems to be shared among many of the C-terminal globular domain mutants.

The existence of Golgi-mediated QC was first suggested by studies in yeast showing that certain mutants of plasma membrane proteins were retained in the Golgi and degraded in the vacuole [46],[47]. Other studies in yeast using heterologous lumenal proteins have implicated a transmembrane protein (Vps10p) as one of the recognition receptors for misfolded proteins within the Golgi [48]. However, analogous Golgi-based QC pathways in mammalian cells have been poorly characterized at present. One example may be the T-cell receptor, whose final assembly occurs within the Golgi. Incomplete TCR complexes have been shown to be retained in the TGN and targeted for lysosomal degradation [49]. Another example may be the Golgi enzyme Furin, aggregates of which are recognized within the Golgi and targeted for degradation in lysosomes [50]. Both of these studies suggested that multimeric status may be a determinant in Golgi-based QC.

Results from our current study are consistent with a similar model, suggesting that PrP may use a more general QC pathway that remains to be studied in detail. Of crucial importance is the machinery and recognition motifs on mutant PrPs used to discriminate folded from misfolded molecules. While additional studies are required to answer this question, the currently available information suggests an intriguing hypothesis. Many PrP mutations may act by favoring dimerization or multimerization of PrP molecules without grossly affecting the overall protein fold. This could explain why ER QC pathways fail to recognize misfolded mutant PrPs. However, this multimerization could create novel motifs (potentially involving the N-terminal lysines) that are recognized by sorting factors in the ER, ERGIC or Golgi, as has been suggested for Furin [50]. This hypothesis is supported by studies using the symmetrical compound, Suramin, whose bivalent interaction with the C-termini of PrP might allow the N-termini to interact with lysosomal routing factors [51]–[53]. The identification of such putative sorting factors is an important goal of our ongoing studies. Misfolded mutant PrPs could serve as an important physiologically relevant model system for dissection of the molecular components of such post-ER QC pathways.

## Materials and Methods

### Constructs, cells and reagents

All PrP constructs are based on human PrP. This is recognized by the 3F4 monoclonal antibody, which does not recognize mouse PrP expressed endogenously by N2a cells. All mutant PrPs were generated by site-directed mutagenesis and the mutation verified by sequencing. The data shown are from human PrPs containing a Valine at position 129, although very similar results were observed for the Methionine variant. Human wtPrP, all mutant PrPs, and fluorescent protein (FP) tagged PrPs were subcloned into and expressed from pCDNA3.1-based vectors (Invitrogen, Carlsbad, CA) by standard procedures. FPs were the monomeric variants of CFP, GFP, and YFP. FP tagged PrPs were created by insertion of PCR amplified FPs (Clontech, Mountain View, CA) into the Bsu36I site in PrP (at residue 50). The monomeric Cerulean and Venus variants of CFP and YFP were used. Co-localization with ER was carried out using GFP-KDEL [54], which contains an ER-targeting signal at the N-terminus, and an ER-retention signal at the C-terminus. Lysosomal co-localization was carried out using either RFP-Lgp-120 (a kind gift from Dr. J. Lippincott-Schwartz) or Lysotracker. TGN and endosomes were visualized using CD-MPR-GFP (a kind gift of Dr. J. Bonifacino). Culture and maintenance of N2a, HeLa, and A4 cells was as described [10],[29],[31]. All experiments involving transient transfections were performed using either Lipofectamine 2000 (Invitrogen) or Effectene (Qiagen, Valencia, CA) as per manufacturer's instructions and cells were analyzed 18–24 hours post-transfection. For Lipofectamine, 2 µg DNA was used per 35 mm dish; for Effectene, 0.4 µg was used. The 3F4 mouse monoclonal antibody against PrP was purchased from Signet Laboratories (Dedham, MA). Brefeldin A, MG132 and Bafilomycin A1 were from EMD Biosciences (La Jolla, CA). Trypsin and Trypsin inhibitor were purchased from Sigma (St. Louis, MO). EndoH and PNGase were from New England Biolabs (Beverly, MA) as were all enzymes used in cloning procedures.

### Biochemical assays

Detergent solubility assays, glycosidase digestions, pulse-chase analysis, immunoblotting, and immunoprecipitations have been described [10],[29],[31]. Briefly, cells were washed with 1× PBS and lysed in ice-cold detergent buffer (DB) containing 150 mM NaCl, 50 mM Tris, pH 7.4, 2 mM EDTA, 0.5% Triton X-100, and 0.5% Deoxycholate. After passage through a 22-guage needle several times, the samples were centrifuged for 30 min at 4°C at 13,000 rpm in a microcentrifuge. Most transfections included ER-localized GFP plasmid (usually comprising one-fifth of the total transfected DNA) to verify that transfection efficiencies were both uniform and greater than 50%. The GFP produced from this marker was also useful in fractionation studies because it served as a transfection control, loading control, and fractionation control: in all experiments, we re-probed the blots with anti-GFP to confirm equal expression, and quantitative recovery in the detergent soluble fraction (data not shown). In addition, all blots were stained for total protein to verify equal loading (data not shown). In experiments where total lysates were analyzed, all cellular material was fully solubilized by boiling in 1% SDS, 0.1 M Tris, pH 8 and processed further for SDS-PAGE. Metabolic labeling, pulse-chase analysis and immunoprecipitations were performed as detailed previously [10],[29],[31]. In experiments where total PrP was being analyzed, cells at each time point were fully solubilized in 1% SDS, 0.1 M Tris, pH 8, boiled, and processed further for immunoprecipitation. In experiments where cells were first fractionated, the cells were harvested in DB, separated into supernatant and pellet fractions, and each fraction was solubilized in SDS, boiled, and immunoprecipitated. The glycan modification of PrP was assessed using EndoH and PNGase digestions on fully denatured cell lysates, as previously described [31]. Where indicated in pulse-chase experiments, inhibitors were added 30 min (10 µg/ml Brefeldin A or 0.1 µg/ml Bafilomycin A1) or 2 hours (5 µM MG132) prior to pulse labeling and maintained during the chase. For steady state inhibitor experiments, inhibitors were maintained in the culture media throughout the experiment. For surface trypsin digestion, cells washed with 1× PBS were incubated for 10 min at 24°C with 100 µg/ml trypsin in NaHM buffer (150 mM NaCl, 20 mM HEPES, 2 mM MgOAc_2_). Trypsin Inhibitor was added to 250 µg/ml, and after 2 min, the cells sedimented by centrifugation, and lysed in detergent as above for further analysis. As a control, detergent (0.2% Triton X-100) was included during the trypsin digestion of a parallel sample to rule out any intrinsic protease resistance of PrP under these conditions. Limited trypsin digestion to assess PrP folding status was performed on total detergent lysates prepared in ice-cold DB. Trypsin was added to the indicated final concentrations from 10 to 100 µg/ml, and incubated for 60 min on ice. Trypsin inhibitor was added to 500 µg/ml, incubated for 5 min on ice, and the samples separated into soluble and insoluble fractions by centrifugation for 30 min in a microcentrifuge at 4°C. 5× SDS-PAGE sample buffer was added to the supernatants and pellets for analysis by immunoblotting. For analysis of total PrP excretion, transfected cells were placed into Opti-MEM media containing or lacking 250 nM Bafilomycin A1 16 h after transfection. After culturing for another 12 h, the conditioned media was collected, debris removed by centrifugation for 2 min in a microcentrifuge at full speed, and the supernatant subjected to TCA precipitation to collect all of the proteins. The precipitated proteins were washed in acetone, and dissolved in SDS-PAGE sample buffer. To isolate exosomes, conditioned media (prepared as above) was centrifuged for 2 min at full speed in a microfuge to remove debris. The supernatant was then subjected to ultracentrifugation at 70,000 rpms in a TLA100.3 rotor with microtest tube adaptors. The pellet containing exosomes was dissolved in SDS-PAGE sample buffer. SDS-PAGE resolution of proteins was performed on 12% Tris-Tricine gels. Quantification was by either phosphorimaging or densitometry. Where densitometry was used, multiple exposures of the gels/blots were obtained and those in the linear range of the film were used. Pulse-chase data was quantified using a Typhoon Phosphorimager and company software (Molecular Dynamics, Sunnyvale, CA) and autoradiographs were digitized using Adobe software (San Jose, CA).

### Fluorescence microscopy and quantitative image analysis

Indirect immunofluorescence and confocal microscopy was as before [29],[31]. Briefly, images were obtained on a confocal microscope (Zeiss LSM510; Carl Zeiss Microimaging, Thornwood, NY) using the manufacturer's image acquisition software. All images were acquired using a 63× oil objective and as 1 Airy unit confocal slices (corresponding to roughly 1 µm thick slice). For indirect immunofluorescent localization of PrP, 3F4 antibody was used at 1∶500 dilution and Alexa-dye conjugated secondary antibody (Alexa-488 or Alexa-546; Invitrogen) was used at 1∶1000 dilution [29]. Single-cell quantitative image analysis is described in detail in Fig. S2. Statistical comparisons between different constructs used the two-tailed Student's t-test. The ratiometric analysis was performed using NIH image software. A macro was written to divide the image into 4×4 pixel regions. The ratio of fluorescence in the green and red channels was calculated for each region containing fluorescence signal above background. This ratio, ranging from 0.1 to 10, was re-scaled to values from 0–255 and plotted into a new image. This ratiometric image was then pseudocolored using the rainbow scale look-up-table.

## Supporting Information

Figure S1 Mutant and wtPrPs occupy heterogeneous but similar cellular locales. (A) Indirect immunofluorescent detection of wtPrP reveals considerable heterogeneity in localization patterns, three of which are shown: predominantly cell surface (left), significant perinuclear in addition to cell surface (middle), and surface, perinuclear, and ER/nuclear envelope. (B) PrP(H187R) analyzed as in panel A also show comparable heterogeneity. The percent intracellular PrP for each cell (quantified in Fig. S2) is indicated in the lower left of the images. (C) Single channel and merge images of cells co-expressing fluorescently tagged wtPrP and PrP(H187R) grown in the presence of 10 µg/ml of Brefeldin A for 8 hours. The two proteins were found to co-localize throughout the ER, without any obvious areas of segregation.
(1.22 MB TIF)

Figure S2 Image analysis for quantitation of total and intracellular PrP. To quantify the surface and intracellular populations of PrP on a per-cell basis, random fields (chosen blindly using a co-transfected RFP marker) of PrP-expressing cells were first imaged under multiple detector conditions (typically three of four) that allow imaging of cells with expression levels spanning ∼25 fold (see example in panel A). Each image is a 1 µm thick confocal section focused at roughly the mid-nuclear level. Each cell was quantified from the respective image in which the fluorescence intensity of that cell falls entirely within the linear range of the detector setting (i.e., the brightest cells are quantified from Exposure 1, and the dimmest from Exposure 4). Regions of interest (see example in panel B) were drawn around the cell periphery (to quantify total fluorescence), and just within the plasma membrane (to quantify intracellular fluorescence). After subtracting background, raw values were obtained for the total, intracellular, and surface (total minus intracellular) fluorescence. These values were normalized for the detector setting used so that values obtained from cells quantified from different exposures could be directly compared. The normalized values were than used to calculate the % intracellular and surface-to-intracellular parameters that are plotted in Fig. 2, 3, and 11. It should be noted that this method of quantification typically underestimates the surface population of PrP relative to the intracellular population. This is because the top and bottom surfaces are never accurately imaged, the latter of which contains substantial surface area. Thus, the average value obtained by imaging gives ∼70–75% surface PrP, while biochemical analyses of the same cell population gives ∼90–95% (as judged by trypsin accessibility). Nonetheless, the single-cell analyses allow direct comparisons to be made among various constructs since the systematic source of error is uniform. Similarly, choosing to quantify a single arbitrary confocal section risks missing significant sources of intracellular fluorescence that are out of the plane of focus. However, this again is a systematic error that is averaged out by the analysis of many cells, and applies uniformly to all constructs analyzed. Thus, as shown in Fig. 2, systematic differences are revealed upon analyses of sufficient cells. More importantly, because expression level data are included in this analyses, the effect of this variable can be analyzed more readily than in biochemical assays performed on cell populations.
(1.68 MB TIF)

Figure S3 Heterogeneity in wtPrP and mutant PrP behavior. (A) Cells were transiently transfected with wtPrP or PrP(E200K) using either Lipofectamine (2 µg DNA) or Effectene (0.4 µg DNA) transfection methods. The soluble (S) and insoluble (P) fraction of the detergent lysates were immunoblotted for PrP. The two blots are taken from the same exposure and processed in parallel, illustrating the markedly different expression levels. The migration of different PrP species are indicated on the left as in Fig. 4. Note that in both cases, increased insoluble, immature species are seen for E200K relative to wtPrP. Parallel blots with an antibody that also recognizes endogenous PrP showed that expression of exogenous PrP transfected with Effectene is comparable to endogenous PrP (data not shown). (B) Cells were transiently co-transfected with GFP and either wtPrP or PrP(H187R) in varying ratios and detergent lysates resolved by SDS-PAGE were immunoblotted for PrP. 1 = Ratio of 4∶1 PrP∶GFP; 2 = Ratio of 2.5∶2.5 PrP∶GFP; 3 = Ratio of 1∶4 PrP∶GFP. The migration of different PrP species is indicated on the left and molecular weight markers are shown on the right. Both faint and dark exposures are shown. Note that while there is an expression level dependent increase in the amount of misfolded (insoluble) PrP in PrP(H187R) expressing cells, this mutant-specific property remains distinguishable at the lowest expression levels, which we estimate to be comparable to normal endogenous PrP. (C) Detergent lysates from cells transiently transfected with wtPrP or PrP(H187R) were immunoblotted for PrP. The left and right panels show results from 2 separate experiments on cultures of different passage numbers to demonstrate that despite heterogeneity between individual experiments, the amount of PrP(H187R) forms recovered from the insoluble fraction was consistently greater than that recovered in the analogous fraction for wtPrP. The most subtle difference we have observed in any experiment is shown in the left panel, while a more typical result is shown in the right panel. Similar heterogeneity was also seen with other mutants. The basis of this heterogeneity remains unclear, and cannot fully be explained by expression level effects alone.
(0.88 MB TIF)

Figure S4 Pulse-chase analysis of PrP(E200K) and PrP(A117V). The metabolism of E200K (panel A) and A117V (panel B) were compared to wtPrP by pulse-chase analysis exactly as in Fig. 7A. Normalized densitometric analysis of the lanes indicated by the asterisks is shown below the autoradiographs. Note that the immature forms are enhanced at the expense of mature forms for E200K. Very similar results were also obtained for D178N (data not shown) and H187R (Fig. 7A). By contrast, A117V looks very similar to wtPrP in its metabolism. Note also that wtPrP looks slightly different in the two experiments (which were done on different days), potentially due to different expression levels (see Fig. S3).
(0.36 MB TIF)
