# Supplementary figures and images for: Selective Processing and Metabolism of Disease-Causing Mutant Prion Proteins
Source: PLoS Pathog. 2009 Jun 19;5(6):e1000479. doi: 10.1371/journal.ppat.1000479 (PMC2691595; doi:10.1371/journal.ppat.1000479)

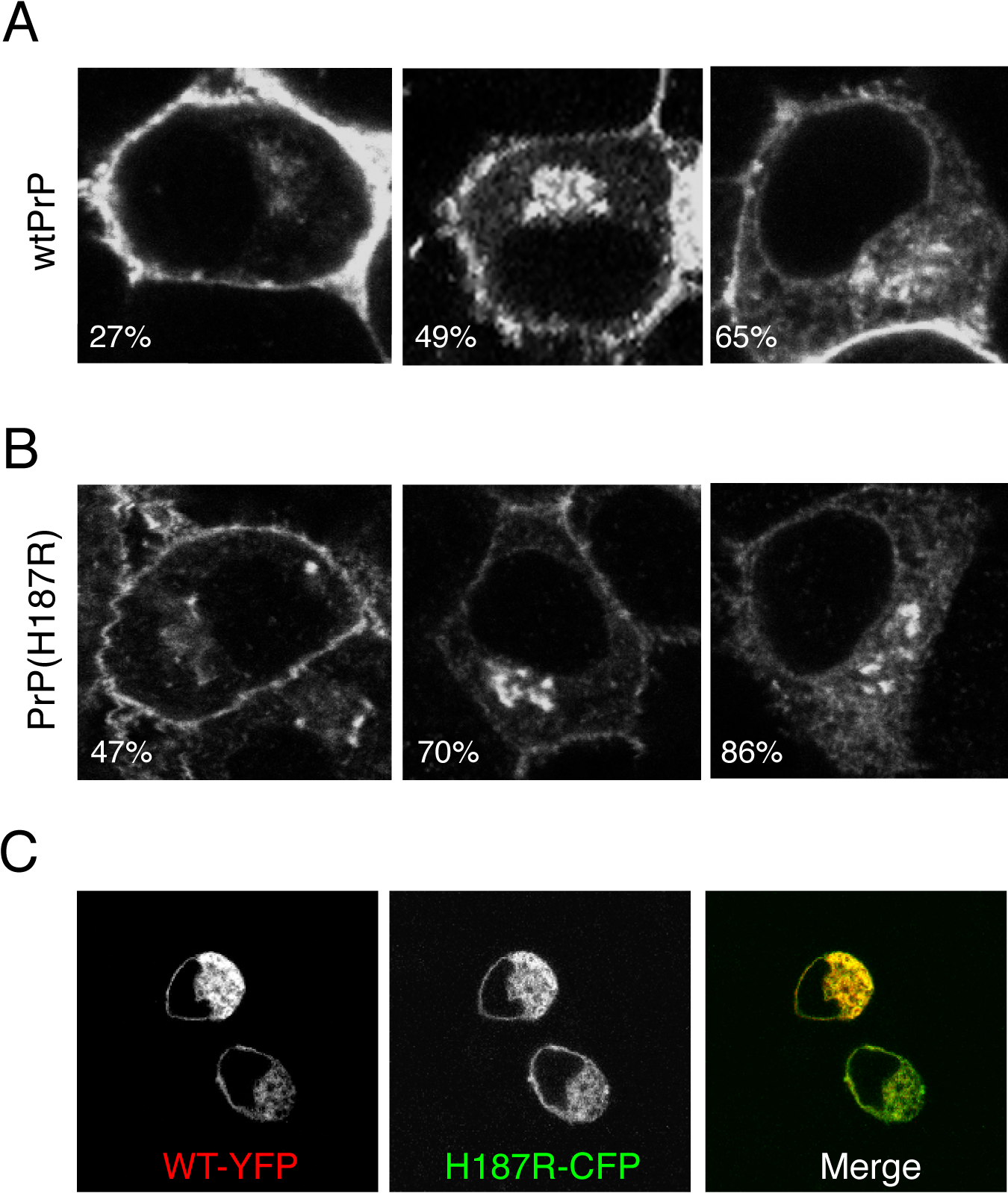

Supplement: Figure S1 — Mutant and wtPrPs occupy heterogeneous but similar cellular locales. (A) Indirect immunofluorescent detection of wtPrP reveals considerable heterogeneity in localization patterns, three of which are shown: predominantly cell surface (left), significant perinuclear in addition to cell surface (middle), and surface, perinuclear, and ER/nuclear envelope. (B) PrP(H187R) analyzed as in panel A also show comparable heterogeneity. The percent intracellular PrP for each cell (quantified in Fig. S2) is indicated in the lower left of the images. (C) Single channel and merge images of cells co-expressing fluorescently tagged wtPrP and PrP(H187R) grown in the presence of 10 µg/ml of Brefeldin A for 8 hours. The two proteins were found to co-localize throughout the ER, without any obvious areas of segregation. (1.22 MB TIF) [file ppat.1000479.s001.tif]

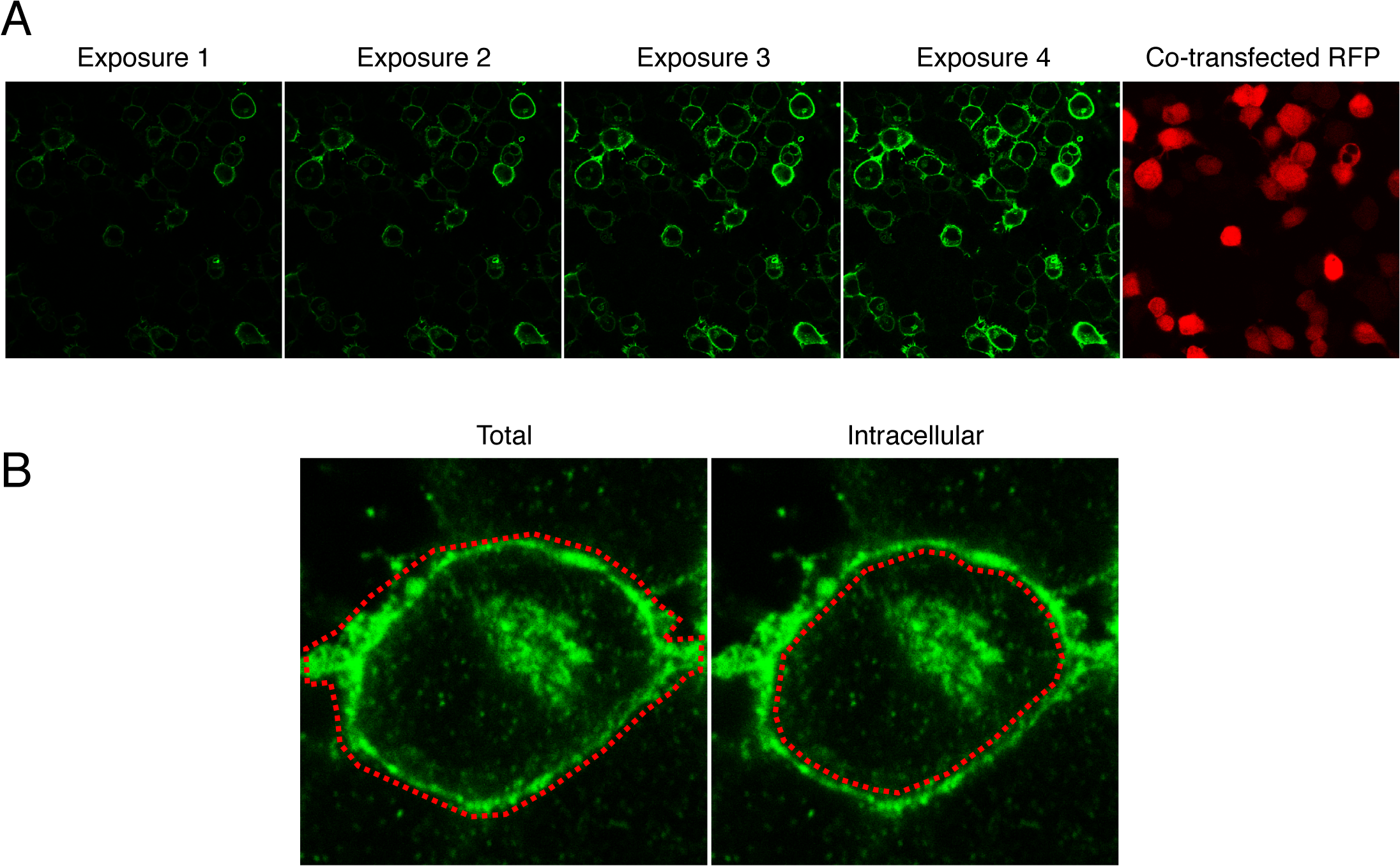

Supplement: Figure S2 — Image analysis for quantitation of total and intracellular PrP. To quantify the surface and intracellular populations of PrP on a per-cell basis, random fields (chosen blindly using a co-transfected RFP marker) of PrP-expressing cells were first imaged under multiple detector conditions (typically three of four) that allow imaging of cells with expression levels spanning ∼25 fold (see example in panel A). Each image is a 1 µm thick confocal section focused at roughly the mid-nuclear level. Each cell was quantified from the respective image in which the fluorescence intensity of that cell falls entirely within the linear range of the detector setting (i.e., the brightest cells are quantified from Exposure 1, and the dimmest from Exposure 4). Regions of interest (see example in panel B) were drawn around the cell periphery (to quantify total fluorescence), and just within the plasma membrane (to quantify intracellular fluorescence). After subtracting background, raw values were obtained for the total, intracellular, and surface (total minus intracellular) fluorescence. These values were normalized for the detector setting used so that values obtained from cells quantified from different exposures could be directly compared. The normalized values were than used to calculate the % intracellular and surface-to-intracellular parameters that are plotted in Fig. 2, 3, and 11. It should be noted that this method of quantification typically underestimates the surface population of PrP relative to the intracellular population. This is because the top and bottom surfaces are never accurately imaged, the latter of which contains substantial surface area. Thus, the average value obtained by imaging gives ∼70–75% surface PrP, while biochemical analyses of the same cell population gives ∼90–95% (as judged by trypsin accessibility). Nonetheless, the single-cell analyses allow direct comparisons to be made among various constructs since the systematic source of error is uniform. Simi [file ppat.1000479.s002.tif]

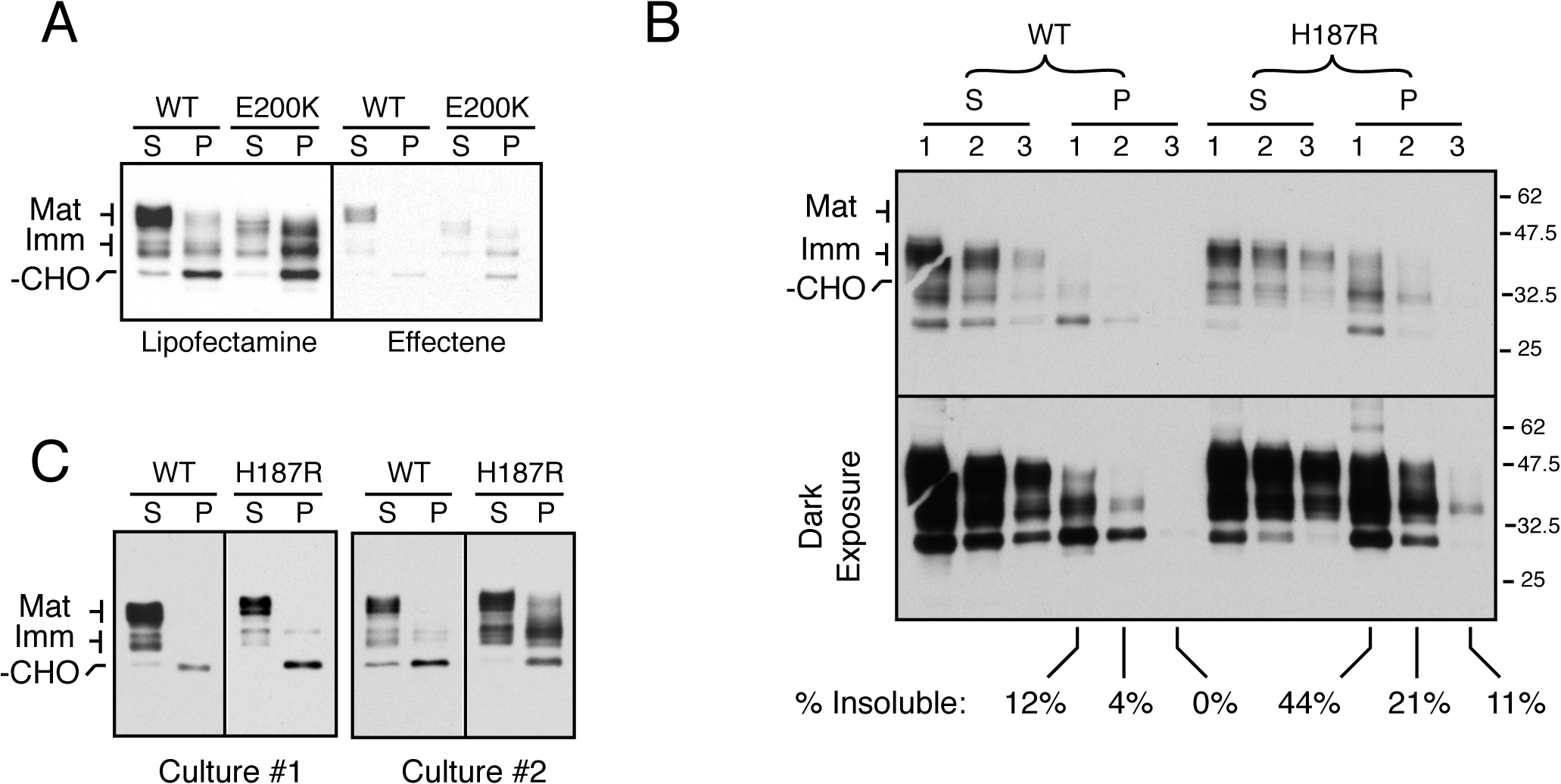

Supplement: Figure S3 — Heterogeneity in wtPrP and mutant PrP behavior. (A) Cells were transiently transfected with wtPrP or PrP(E200K) using either Lipofectamine (2 µg DNA) or Effectene (0.4 µg DNA) transfection methods. The soluble (S) and insoluble (P) fraction of the detergent lysates were immunoblotted for PrP. The two blots are taken from the same exposure and processed in parallel, illustrating the markedly different expression levels. The migration of different PrP species are indicated on the left as in Fig. 4. Note that in both cases, increased insoluble, immature species are seen for E200K relative to wtPrP. Parallel blots with an antibody that also recognizes endogenous PrP showed that expression of exogenous PrP transfected with Effectene is comparable to endogenous PrP (data not shown). (B) Cells were transiently co-transfected with GFP and either wtPrP or PrP(H187R) in varying ratios and detergent lysates resolved by SDS-PAGE were immunoblotted for PrP. 1 = Ratio of 4∶1 PrP∶GFP; 2 = Ratio of 2.5∶2.5 PrP∶GFP; 3 = Ratio of 1∶4 PrP∶GFP. The migration of different PrP species is indicated on the left and molecular weight markers are shown on the right. Both faint and dark exposures are shown. Note that while there is an expression level dependent increase in the amount of misfolded (insoluble) PrP in PrP(H187R) expressing cells, this mutant-specific property remains distinguishable at the lowest expression levels, which we estimate to be comparable to normal endogenous PrP. (C) Detergent lysates from cells transiently transfected with wtPrP or PrP(H187R) were immunoblotted for PrP. The left and right panels show results from 2 separate experiments on cultures of different passage numbers to demonstrate that despite heterogeneity between individual experiments, the amount of PrP(H187R) forms recovered from the insoluble fraction was consistently greater than that recovered in the analogous fraction for wtPrP. The most subtle difference we have observed in any experiment is shown [file ppat.1000479.s003.tif]

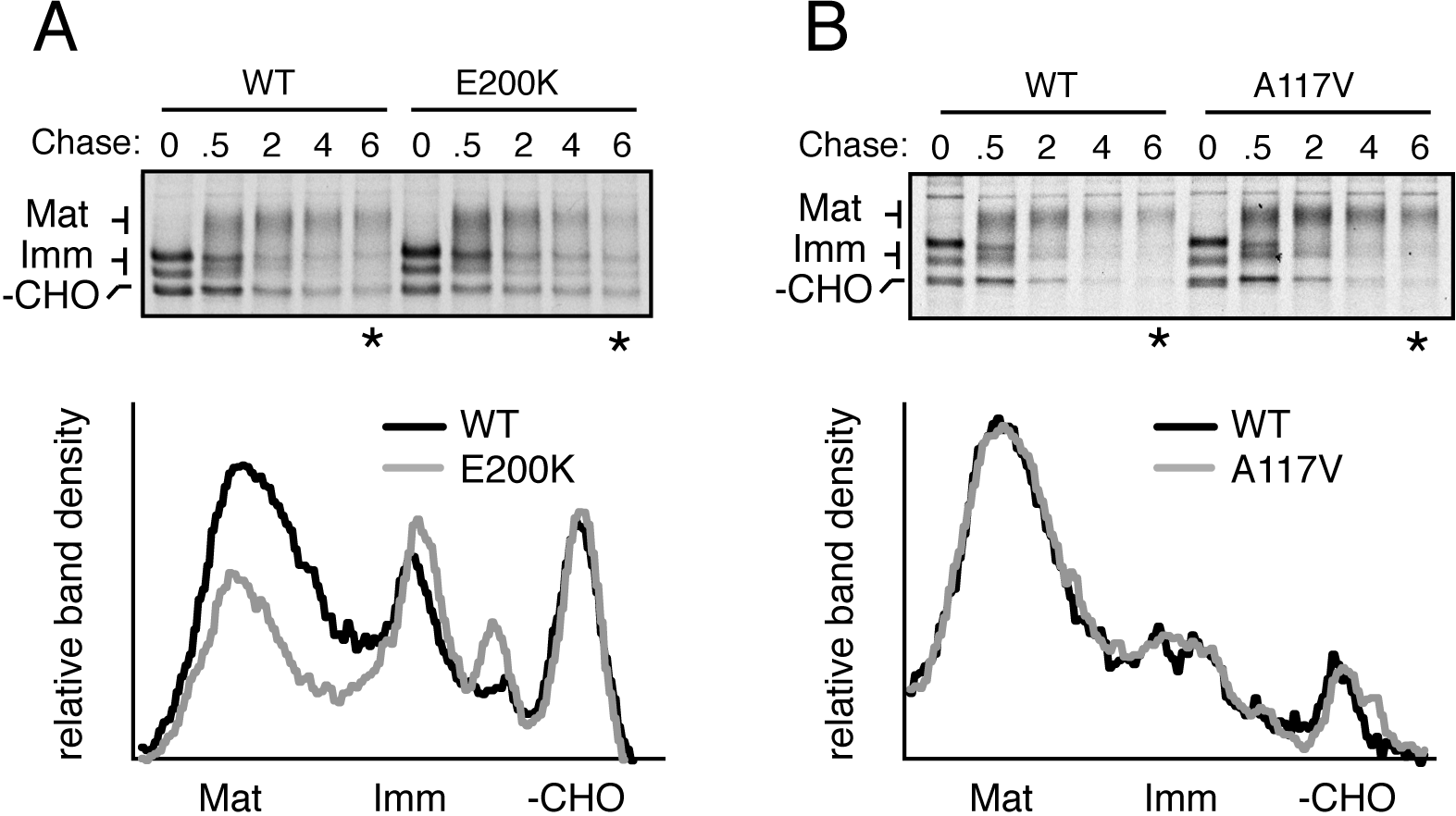

Supplement: Figure S4 — Pulse-chase analysis of PrP(E200K) and PrP(A117V). The metabolism of E200K (panel A) and A117V (panel B) were compared to wtPrP by pulse-chase analysis exactly as in Fig. 7A. Normalized densitometric analysis of the lanes indicated by the asterisks is shown below the autoradiographs. Note that the immature forms are enhanced at the expense of mature forms for E200K. Very similar results were also obtained for D178N (data not shown) and H187R (Fig. 7A). By contrast, A117V looks very similar to wtPrP in its metabolism. Note also that wtPrP looks slightly different in the two experiments (which were done on different days), potentially due to different expression levels (see Fig. S3). (0.36 MB TIF) [file ppat.1000479.s004.tif]
